# Assessing the Relative Importance of Imaging and Serum Biomarkers in Capturing Disability, Cognitive Impairment, and Clinical Progression in Multiple Sclerosis

**DOI:** 10.1002/advs.202512946

**Published:** 2026-01-12

**Authors:** Alessandro Cagol, Pascal Benkert, Sabine Schaedelin, Mario Ocampo‐Pineda, Noemi Montobbio, Po‐Jui Lu, Batuhan Ayci, Antonia Wenger, Alfi Aran Shukur, Kornelius Kaim, Lester Melie‐Garcia, Matthias Weigel, Alessio Signori, Pasquale Calabrese, Ludwig Kappos, Maria Pia Sormani, Jens Kuhle, Cristina Granziera

**Affiliations:** ^1^ Translational Imaging in Neurology (ThINk) Basel, Department of Biomedical Engineering Faculty of Medicine University Hospital Basel and University of Basel Basel Switzerland; ^2^ Multiple Sclerosis Centre, Departments of Neurology Clinical Research and Biomedicine University Hospital and University Basel Basel Switzerland; ^3^ Research Center for Clinical Neuroimmunology and Neuroscience Basel (RC2NB) University Hospital Basel and University of Basel Basel Switzerland; ^4^ Dipartimento di Scienze della Salute Università degli Studi di Genova Genova Italy; ^5^ Department of Clinical Research University Hospital Basel University of Basel Basel Switzerland; ^6^ Cerrahpasa Medical School Istanbul University‐Cerrahpasa Istanbul Turkey; ^7^ Division of Radiological Physics Department of Radiology University Hospital Basel Basel Switzerland; ^8^ Neuropsychology and Behavioral Neurology Unit Division of Cognitive and Molecular Neuroscience University of Basel Basel Switzerland; ^9^ IRCCS Ospedale Policlinico San Martino Genova Italy

**Keywords:** MRI, Multiple Sclerosis, Progression Independent of Relapse Activity, Quantitative MRI, Serum Glial Fibrillary Acidic Protein, Serum Neurofilament Light Chain, Spinal Cord Atrophy

## Abstract

The heterogeneity of multiple sclerosis (MS) pathology calls for robust biomarkers to predict disability and progression, particularly progression independent of relapse activity (PIRA). Here, we aimed to identify the most informative MRI and serum biomarkers for predicting clinical outcomes in people with MS (pwMS), including disability severity, cognitive impairment, disease phenotype, and risk of PIRA. We applied a machine learning–based feature selection approach to cross‐sectional and longitudinal data from two independent pwMS cohorts. Cohort 1 (n = 120) included 57 MRI biomarkers, incorporating advanced quantitative MRI (qMRI). Cohort 2 (n = 279) included 35 MRI biomarkers derived from conventional MRI. Both cohorts obtained serum neurofilament light chain (sNfL) and glial fibrillary acidic protein (sGFAP) measurements. Spinal cord atrophy consistently emerged as the strongest predictor of disability severity and predicted PIRA, along with cortical thinning and subcortical atrophy – particularly in deep gray matter. sNfL, sGFAP, and qMRI metrics independently contributed to the prediction of PIRA and progressive disease phenotype. In conclusion, our findings show that spinal cord atrophy and cortical degeneration are the most robust and consistent predictors of MS severity and progression. Serum biomarkers of neuroaxonal and astrocytic damage, together with qMRI‐derived tissue metrics, provide independent and complementary value for outcome prediction.

## Graphical abstract

Using machine‐learning analyses in two independent multiple sclerosis cohorts, spinal cord atrophy and cortical degeneration emerged as key predictors of disability and progression independent of relapses. Deep gray matter damage further improved prediction, while serum biomarkers of brain damage provided complementary information, highlighting the value of a multimodal approach to stratify disease severity and progression risk.

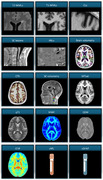

## 1 Introduction

Multiple sclerosis (MS) is a chronic condition characterized by demyelination, inflammation, and neurodegeneration within the central nervous system (CNS). Magnetic resonance imaging (MRI) plays a pivotal role in the diagnosis and clinical management of people with MS (pwMS), primarily by enabling the detection of white matter lesions (WMLs). However, despite the recognized importance of WML assessment in clinical practice, the correlation between WML burden and clinical symptoms is often weak – a phenomenon referred to as the *clinico‐radiological paradox* [1]. This discrepancy stems from several factors, encompassing the limited pathological specificity of conventional MRI, the frequent oversight of spinal cord involvement, and the insensitivity of standard imaging techniques to diffuse damage in normal‐appearing tissue [1]. Additionally, reparative mechanisms and adaptive processes largely go undetected by conventional MRI.

These limitations have driven the search for biomarkers with greater sensitivity and specificity to quantify pathological changes in pwMS. Significant progress has been made in characterizing focal damage, particularly through the development of techniques to identify cortical lesions (CLs), which are closely associated with physical and cognitive impairment, as well as disease progression [2, 3]. Advanced imaging techniques have also emerged to capture the heterogeneity of WMLs in terms of demyelination, neuroaxonal loss, smoldering inflammation, and repair. For instance, the detection of paramagnetic rim lesions (PRLs) has enabled the identification of chronic active lesions, which are characterized by persistent inflammatory activity and are linked to worse clinical outcomes [4, 5]. Furthermore, quantitative MRI (qMRI) has enriched our understanding of MS pathology by offering microstructural insights into tissue changes and providing proxy measures of demyelination, macromolecular loss, neuroaxonal degeneration, and iron dysregulation [6]. These metrics are valuable for characterizing WMLs and detecting subtle abnormalities in tissue that appears normal on conventional MRI [7]. In addition, brain volumetric measurements have also become a widely used research tool for quantifying tissue loss, serving as a surrogate marker for the overall neurodegenerative burden [8]. Improved techniques for assessing spinal cord pathology, both in terms of focal lesions and diffuse tissue damage, have similarly been developed [8]. Beyond imaging, serum biomarkers such as neurofilament light chain (sNfL) and glial fibrillary acidic protein (sGFAP) have demonstrated significant potential in capturing inflammatory and neurodegenerative processes. While sNfL primarily reflects axonal damage, sGFAP serves as a marker of astrocytic activation, and both have been shown to correlate with clinical severity and predict disease progression [9, 10, 11].

These advancements have expanded our knowledge of MS pathology and provided a broad array of potential biomarkers for use in clinical trials and practice. However, most studies to date have focused on a limited subset of these biomarkers, leaving gaps in our understanding of their relative contributions. Moreover, implementing all these biomarkers in clinical practice is impractical and could lead to redundancy. Thus, it is essential to identify the most relevant biomarkers for disease management, monitoring, and treatment decisions to optimize care for pwMS. This is particularly critical for developing biomarkers that predict disease evolution, especially in addressing the neurodegenerative component of MS, which is considered the primary driver of progression independent of relapse activity (PIRA) [12]. Tackling PIRA remains one of the most pressing unmet needs for improving long‐term outcomes in pwMS.

In this context, we conducted a cross‐sectional and longitudinal study to assess the relative contribution of a wide range of MRI biomarkers reflecting brain and spinal cord pathology, along with serum biomarkers, in explaining clinical outcomes in pwMS. Our analysis incorporated both conventional and advanced MRI metrics, capturing focal and diffuse inflammatory as well as neurodegenerative changes at macroscopic and microstructural levels. To identify the most informative biomarkers, we applied a data‐driven machine learning approach designed to robustly capture the relative importance of each feature in explaining clinical outcomes. Specifically, we aimed to determine the extent to which each biomarker contributes to explaining: (1) the severity of neurological disability, (2) the degree of cognitive impairment, (3) the clinical disease phenotype, and (4) the risk of future disease progression due to PIRA. Associations of a subset of the selected biomarkers with outcomes (1), (3), and (4) were further evaluated in an independent validation cohort.

## 2 Methods

### 2.1 Participants and Study Design

In this longitudinal, prospective observational study, we used data from two independent cohorts. Cohort 1 included both pwMS and healthy controls (HCs) who underwent advanced MRI and serum assessments at baseline, along with longitudinal clinical follow‐up. Cohort 2 included pwMS who underwent conventional MRI and serum assessments at baseline, also followed by longitudinal clinical observation.

**FIGURE 1:**
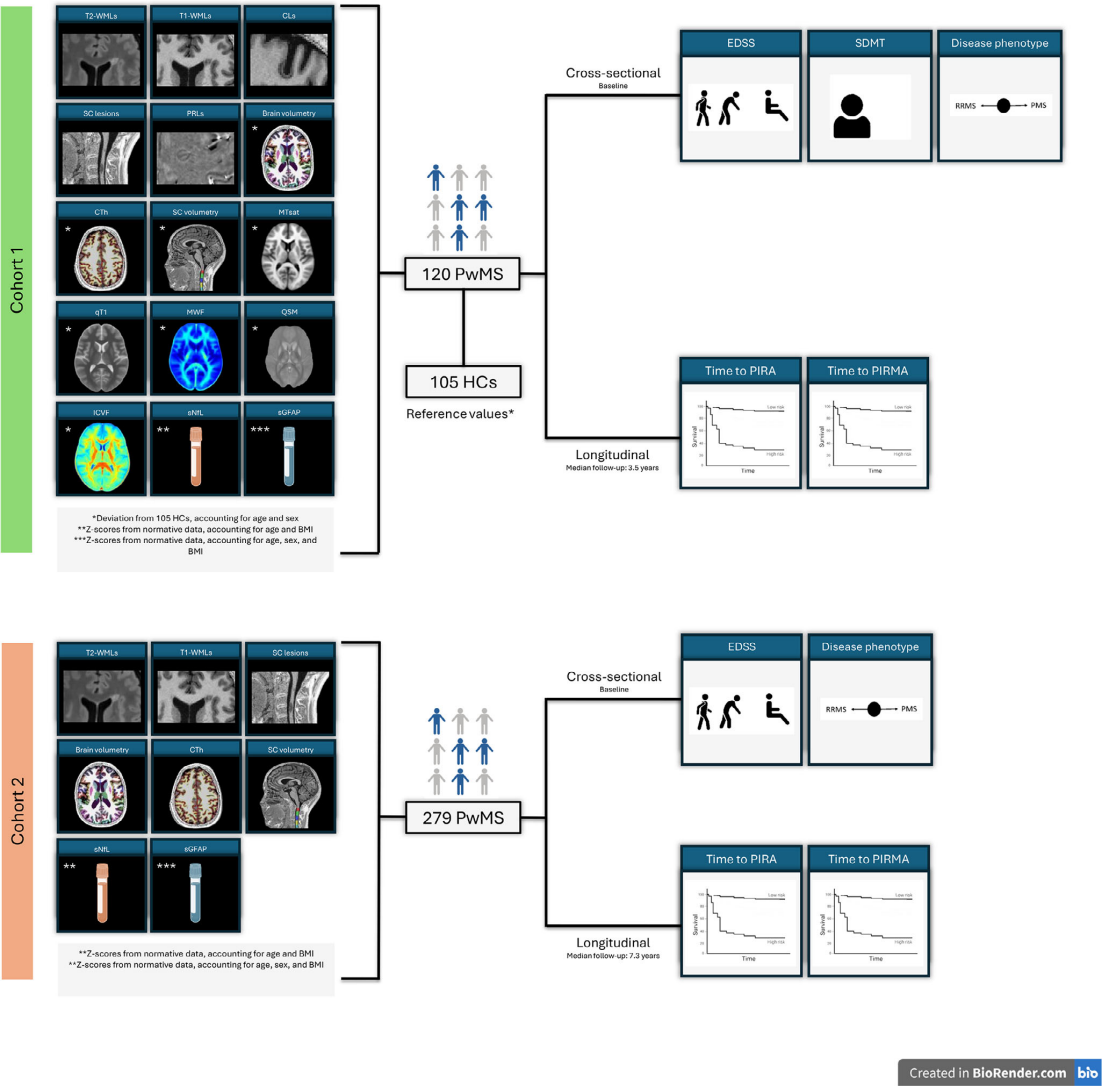
Study design. Two independent cohorts of people with multiple sclerosis (PwMS) were analyzed. Cohort 1 included extensive multimodal MRI and serum biomarkers and was used for cross‐sectional analyses of disability and disease phenotype, as well as longitudinal analyses of time to progression independent of relapse activity (PIRA) and progression independent of relapse and MRI activity (PIRMA). Cohort 2 included a more limited set of clinically available MRI and serum measures and was used for replication and validation analyses. Cross‐sectional outcomes were assessed at baseline, while longitudinal outcomes were evaluated over follow‐up. Abbreviations: CLs, cortical lesions; CSA, cross‐sectional area; CTh, cortical thickness; EDSS, Expanded Disability Status Scale; HCs, healthy controls; ICVF, intracellular volume fraction; MTsat, magnetization transfer saturation; MWF, myelin water fraction; PIRMA, progression independent of relapse and MRI activity; PIRA, progression independent of relapse activity; PMS, progressive multiple sclerosis; PRLs, paramagnetic rim lesions; PwMS, people with multiple sclerosis; qT1, quantitative T1; QSM, quantitative susceptibility mapping; RRMS, relapsing–remitting multiple sclerosis; SC, spinal cord; SDMT, Symbol Digit Modalities Test; sGFAP, serum glial fibrillary acidic protein; sNfL, serum neurofilament light chain; T1‐WMLs, T1‐weighted white matter lesions; T2‐WMLs, T2‐weighted white matter lesions.

The study was approved by the local ethics committee, and all patients provided written informed consent before study entry. An overview of the study design is provided in Figure 1.

This study followed the STROBE reporting guideline.

#### 2.1.1 Cohort 1

Cohort 1 included pwMS who participated in both the INsIDER study (*NCT05177523*) and the Swiss Multiple Sclerosis Cohort (SMSC) study [13]. Inclusion criteria for pwMS were as follows:

1. Availability of an advanced brain MRI scan conducted as part of the INsIDER study. The MRI scan date served as the study baseline for each participant.
2. Availability of at least two clinical follow‐up examinations, each conducted at least six months apart from one another and from the study baseline, performed prospectively as part of the SMSC study protocol.
3. Diagnosis of MS according to the 2017 revised McDonald criteria [14].
4. Age between 18 and 80 years.
5. Absence of relevant neurological or psychiatric comorbidities.

Additionally, Cohort 1 included HCs participating in the INsIDER study, with the availability of an advanced brain MRI scan obtained using the same protocol as in pwMS.

Exclusion criteria for all participants encompassed pregnancy, contraindications to MRI, inability to provide informed consent, relapses or steroid treatment within the prior 3 months, and insufficient MRI quality. All eligible participants were enrolled.

#### 2.1.2 Cohort 2

Cohort 2 included pwMS participating in the SMSC study, followed at the University Hospital of Basel, and meeting the following inclusion criteria:

1. Availability of a 3‐Tesla (3T) brain MRI scan conducted as part of the SMSC study. The date of the first available 3T MRI scan served as the study baseline for each participant.
2. Availability of at least two clinical follow‐up examinations, each conducted at least six months apart from one another and from the study baseline, performed prospectively as part of the SMSC study protocol.
3. Diagnosis of MS according to the 2017 revised McDonald criteria [14].
4. Age between 18 and 80 years.
5. Absence of relevant neurological or psychiatric comorbidities.

Exclusion criteria were identical to those applied in Cohort 1.

### 2.2 Clinical Data

All participants in both cohorts underwent regular clinical evaluations, conducted at least annually, as part of the SMSC study [13]. Standardized assessments included the calculation of the Expanded Disability Status Scale (EDSS) score (https://www.neurostatus.net/) by certified raters.

The occurrence of PIRA during follow‐up was defined as an increase in EDSS score (≥1.5, ≥1.0, or ≥0.5 points if baseline EDSS was 0, 1.0–5.5, or >5.5, respectively) using a roving baseline [15], confirmed after at least 6 months, in the absence of relapses between the EDSS increase and the preceding reference visit (conducted ≥90 days before the EDSS increase) and between the EDSS increase and its confirmation [16]. PIRA events were identified using the *msprog* package [17].

In Cohort 1, cognitive performance was assessed using the oral version of the Symbol Digit Modalities Test (SDMT) in a subset of 97 pwMS and 100 HCs, with raw SDMT scores converted to z‐scores based on normative data [18].

### 2.3 MRI Acquisition and Analysis

For Cohort 1, advanced brain MRI examinations were conducted using a standardized acquisition protocol on a 3T whole‐body MR system (*Magnetom Prisma, Siemens Healthineers*), using a 64‐channel phased‐array head and neck coil for radiofrequency reception. The protocol included: (1) 3D fluid‐attenuated inversion recovery (FLAIR), (2) 3D magnetization‐prepared 2 rapid gradient‐echo (MP2RAGE), (3) multi‐shell diffusion, (4) 3D echo planar imaging (EPI), (5) three 3D radiofrequency spoiled gradient echo acquisitions used to obtain magnetization transfer saturation (MTsat) maps [19], and (6) fast acquisition with spiral trajectory and adiabatic T2‐prep (FAST‐T2). FLAIR and MP2RAGE images covered the spinal cord from C1 to C4.

The standardized brain MRI protocol for Cohort 2 included: (1) 3D FLAIR, and (2) 3D magnetization‐prepared rapid gradient‐echo (MPRAGE), both covering the spinal cord from C1 to C4. Further protocol details are provided in Tables S1 and S2.

Due to differences in MRI acquisition protocols, only a subset of MRI biomarkers could be quantified in Cohort 2 (Figure 1).

#### 2.3.1 Lesion Segmentation

T2‐hyperintense white matter lesions (T2‐WMLs) were segmented using a deep learning‐based tool [20] and manually refined. Cortical lesions (CLs) were manually segmented on MP2RAGE by two expert raters [21]. T1‐hypointense WMLs (T1‐WMLs) were automatically segmented on MP2RAGE/MPRAGE using *SAMSEG (v7.2.0)* [22]. PRLs, defined as discrete FLAIR‐hyperintense lesions either completely or partially encircled by a rim of paramagnetic signal, visible in at least one contrast between unwrapped phase and quantitative susceptibility mapping (QSM) [23], were manually detected by two expert raters. Spinal cord lesions in the C1‐C4 tract were manually identified on FLAIR and MP2RAGE/MPRAGE images.

#### 2.3.2 Brain Morphometry

Segmentation of brain structures and calculation of global/regional brain volumes and cortical thickness (CTh) were performed with *FreeSurfer (v.6.0.0;* http://surfer.nmr.mgh.harvard.edu/), with lesion‐filled MP2RAGE/MPRAGE images as input. Results were manually checked and edited as needed. Brain volumes were normalized by the total intracranial volume (TIV) obtained with *SAMSEG* [24].

#### 2.3.3 Spinal Cord Morphometry

The mean cross‐sectional area (CSA) at C1, C2, C3, and C4 was measured using *Spinal Cord Toolbox* [25] *(v.5.3.0)* on MP2RAGE/MPRAGE images. Manual labelling of intervertebral discs was performed to ensure accuracy, and outputs were manually checked.

#### 2.3.4 qMRI Analysis

We analyzed 5 different qMRI contrasts providing insights into: (1) micro/macrostructural integrity (quantitative T1 [qT1]), (2) macromolecular content (MTsat), (3) myelin content (myelin water fraction [MWF]), (4) axon and dendrite density (intracellular volume fraction [ICVF]), and (5) iron and myelin content (QSM) [6]. qT1 maps were derived from MP2RAGE images [26]. MTsat maps were reconstructed using magnetization transfer‐weighted, proton density‐weighted, and T1‐weighted images [19]. MWF maps were generated from FAST‐T2 data [27]. ICVF values were estimated using Neurite Orientation Dispersion and Density Imaging (NODDI) applied to diffusion images [28], after denoising and correction for motion, geometric distortions, and eddy‐currents. QSM maps were reconstructed from 3D‐EPI images using the morphology‐enabled dipole inversion (MEDI) algorithm [29].

Mean qMRI values were extracted within WMLs, normal‐appearing white matter (NAWM), normal‐appearing cortical and deep gray matter (GM), and the normal‐appearing thalamus.

#### 2.3.5 Longitudinal WML Changes

All longitudinal conventional MRI scans acquired as part of the SMSC study during the clinical follow‐up were analyzed to identify new or enlarging T2‐WMLs. These lesions were automatically detected [30] and manually verified to ensure accuracy. The identification of new or enlarging WMLs was instrumental in determining who among pwMS experienced progression independent of relapse and MRI activity (PIRMA), defined as PIRA occurring in the absence of MRI‐detected inflammatory activity [12, 16]. The protocol details for conventional MRI scans are provided in Table S2.

### 2.4 Neurofilament Light Chain

sNfL and sGFAP levels were quantified using the ultrasensitive single molecule array (Simoa) technology (Quanterix), following the manufacturer's protocol [10]. Z‐scores were calculated using normative reference data, accounting for age and body mass index (BMI) for sNfL, and for age, BMI, and sex for sGFAP [31].

### 2.5 Statistical Analysis

All statistical analyses were conducted using *R* (*version 4.3.1*), with statistical significance set at p<0.05.

The Boruta algorithm (configured with 10,000 trees, 2,000 iterations) [32, 33], an extension of the random forest algorithm, was employed to evaluate the contribution of each biomarker in explaining neurological disability, cognitive performance, disease phenotype, and occurrence of PIRA. The Boruta algorithm is a feature selection method that compares the importance of original variables against randomly permuted “shadow” variables. By iteratively fitting random forest models, it evaluates feature importance by comparing it to the highest importance achieved by the shadow variables.

In Cohort 1, we included as predictors in the Boruta models clinical and demographic factors, brain and spinal cord volumetric measurements, qMRI metrics, measures of lesion burden, and serum biomarkers. A comprehensive list of the 59 included predictors is available in Table S3. To account for the linear dependence of brain and spinal cord volumetric measurements and qMRI metrics on age and sex, these variables were adjusted by calculating their deviation from the HC population. Linear regressions were fitted within the HC population with volumetric or qMRI metrics as the dependent variables and age and sex as explanatory variables. The residuals from these regressions in pwMS, representing deviations from expected values, were used as input variables in the Boruta models [34]. Age and sex were also included as predictors in the models to minimize potential remaining confounding effects.

Separate Boruta models were applied to the following dependent variables: (1) EDSS, analyzed both as a continuous variable and dichotomized based on a cut‐off of either ≥3.0 or ≥6.0; (2) SDMT z‐score; (3) disease phenotype (PMS vs. RRMS); and (4) PIRA incidence. For PIRA, we used a survival model to analyze time‐to‐event data and additionally assessed PIRA as a binary outcome. To account for potential baseline differences in demographic and clinical variables between patients with and without PIRA, 1:1 nearest‐neighbor propensity score matching was conducted. The matching criteria included age, sex, disease duration, EDSS, disease‐modifying treatment (DMT) class, disease phenotype, and follow‐up duration.

The predictive performance of variables selected by the Boruta models was assessed using random forest models with tenfold cross‐validation. Performance metrics included mean R^2^ for regression tasks, mean area under the receiver operating characteristic curve (AUC) for classification tasks, and mean concordance index (C‐index) for survival analysis.

Sensitivity and complementary analyses were conducted to address methodological limitations, improve interpretability, and further characterize model performance. Because variable‐importance estimates in the Boruta model may be affected by strong collinearity among predictors, we conducted sensitivity analyses using conditional Boruta models implemented with conditional random forests (*cforest*, party *R* package), which estimate conditional variable importance while accounting for correlations between predictors. To further evaluate the temporal stability of PIRA prediction, model performance was additionally quantified using time‐dependent AUCs calculated at multiple follow‐up time intervals. To improve interpretability of the PIRA prediction models, we explored post hoc explanation approaches based on Shapley additive explanations, including SHAP dependence plots, which describe both the relative importance and the directionality of individual predictors in the random forest models. In parallel, we fitted linear survival models using ridge‐penalized Cox regression as a complementary, more interpretable modeling framework.

Additional sensitivity analyses included: (1) recalculating SDMT z‐scores based on the HC population rather than normative data, (2) including EDSS as an additional explanatory variable in the model predicting time to PIRA, (3) including DMT class as an additional explanatory variable in the model predicting time to PIRA, (4) analyzing time to PIRA specifically within the RRMS patient group, and (5) investigating time to PIRMA.

Analogous Boruta models, implemented with the same hyperparameters, were applied to the validation cohort (Cohort 2) to assess the relative importance of biomarkers. In this cohort, data were available for a subset of 37 out of the 59 biomarkers used in Cohort 1, as detailed in Table S4. Due to the absence of a paired HC cohort, biomarkers were entered into the models using their original values; all models included age, sex, and disease duration as covariates. As in Cohort 1, outcomes of interest included cross‐sectional EDSS and disease phenotype at baseline, as well as prediction of future time to PIRA and discrimination between propensity score‐matched pwMS with and without PIRA during follow‐up. Matching criteria were identical to those applied in Cohort 1. To assess clinical applicability, we derived a parsimonious risk score using ridge regression based on clinically accessible biomarkers identified as predictors of PIRA by the Boruta analysis in Cohort 2; this score was subsequently validated in Cohort 1.

Additional methodological details are available in Supporting Methods.

## 3 Results

### 3.1 Cohort 1

**FIGURE 2:**
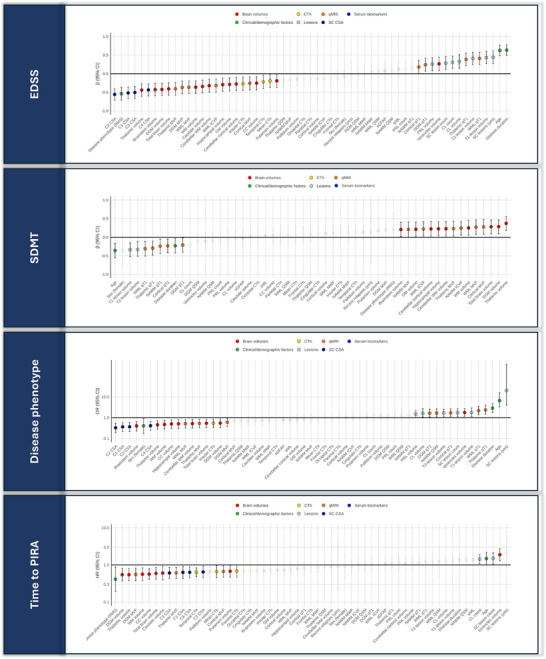
Univariate associations in Cohort 1. Abbreviations: CC, corpus callosum; CI, confidence interval; CL, cortical lesion; CSA, cross‐sectional area; CTh, cortical thickness; DGM, deep gray matter; EDSS, Expanded Disability Status Scale; GM, gray matter; HR, hazard ratio; ICVF, intracellular volume fraction; MVF, myelin volume fraction from magnetization transfer saturation; MWF, myelin water fraction; NAWM, normal‐appearing white matter; PIRA, progression independent of relapse activity; PRL, paramagnetic rim lesions; qMRI, quantitative MRI; QSM, quantitative susceptibility mapping; qT1, quantitative T1; RMS, relapsing multiple sclerosis; SC, spinal cord; SDMT, Symbol Digit Modalities Test; sGFAP, serum glial fibrillary acidic protein; sNfL, serum neurofilament light chain; WM, white matter; WML, white matter lesions.

A total of 120 pwMS and 105 HCs (55% female; mean age: 37.8 (SD: 13.0) years) were included in Cohort 1. Key characteristics of pwMS are provided in Table 1. The median follow‐up time was 3.5 years (IQR: 2.6; 4.2). Univariate associations between variables used in the Boruta models and outcome measures (i.e. EDSS, SDMT, disease phenotype, and time to PIRA) are visually represented in Figure 2.

**TABLE 1:** Key characteristics of pwMS.

	Cohort 1	Cohort 2
Participants, No.	120	279
Females, No. (%)	70 (58.3)	200 (71.7)
Age, mean (SD), years	47.7 (13.6)	45.6 (11.6)
Disease duration, median [IQR], years	12.4 [4.2; 24.5]	10.2 [6.0; 17.8]
Disease phenotype:		
‐ RRMS	89 (74.2)	259 (92.8)
‐ SPMS	20 (16.7)	10 (3.6)
‐ PPMS	11 (9.2)	10 (3.6)
DMT: ‐ Platform treatments, No. (%)	5 (4.2)	26 (9.3)
‐ Oral treatments, No. (%)	40 (33.3)	160 (57.3)
‐ Monoclonal antibody treatments, No. (%)	57 (47.5)	47 (16.8)
‐ Untreated, No. (%)	18 (15.0)	46 (16.5)
EDSS, median [IQR]	3.0 [2.0; 5.0]	2.0 [1.5; 3.25]
sNfL Z‐score, median [IQR]	0.39 [‐0.36; 1.08]	0.23 [‐0.63; 0.99]
sGFAP Z‐ascore, median [IQR]	0.14 [‐0.71; 1.00]	0.00 [‐0.71; 0.60]
T2LV, median [IQR], mL	7.2 [2.8; 16.3]	5.6 [2.2; 14.1]
Cortical lesion count, median [IQR]	2 [0; 5.5]	/
PRL count, median [IQR]	3 [0.3; 6.0]	/
C1‐C4 lesion count, median [IQR]	1 [0; 2.3]	3 [3; 5]
Normalized brain volume, median [IQR]	0.70 [0.66; 0.74]	0.69 [0.66; 0.72]
Normalized WM volume, median [IQR]	0.29 [0.27; 0.30]	0.28 [0.27; 0.30]
Normalized GM volume, median [IQR]	0.40 [0.37; 0.42]	0.38 [0.36; 0.41]
Normalized cortical volume, median [IQR]	0.30 [0.27; 0.32]	0.29 [0.20; 0.30]
Normalized DGM volume, median [IQR]	0.03 [0.03; 0.04]	0.03 [0.03; 0.04]
CTh, median [IQR], mm	2.33 [2.2; 2.41]	2.38 [2.28; 2.45]
C2‐C3 CSA, median [IQR], mm^2^	60.2 [53.3; 65.6]	60.8 [55.7; 65.9]
Follow‐up duration, median [IQR], years	3.5 [2.6; 4.2]	7.3 [6.7; 7.9]
Patients exhibiting PIRA during follow‐up, No. (%)	28 (23.3)	79 (28.3)

Abbreviations: CTh = cortical thickness; DMT = disease modifying treatment ; EDSS = Expanded Disability Status Scale; GM = gray matter; IQR = interquartile range; PIRA = progression independent of relapse activity; PPMS = primary progressive multiple sclerosis; PRL = paramagnetic rim lesions; pwMS = people with multiple sclerosis; RRMS = relapsing‐remitting multiple sclerosis; SD = standard deviation; sNfL = serum neurofilament light chain; SPMS = secondary progressive multiple sclerosis; T2LV = T2‐lesion volume; WM = white matter.

#### 3.1.1 EDSS Prediction

**FIGURE 3:**
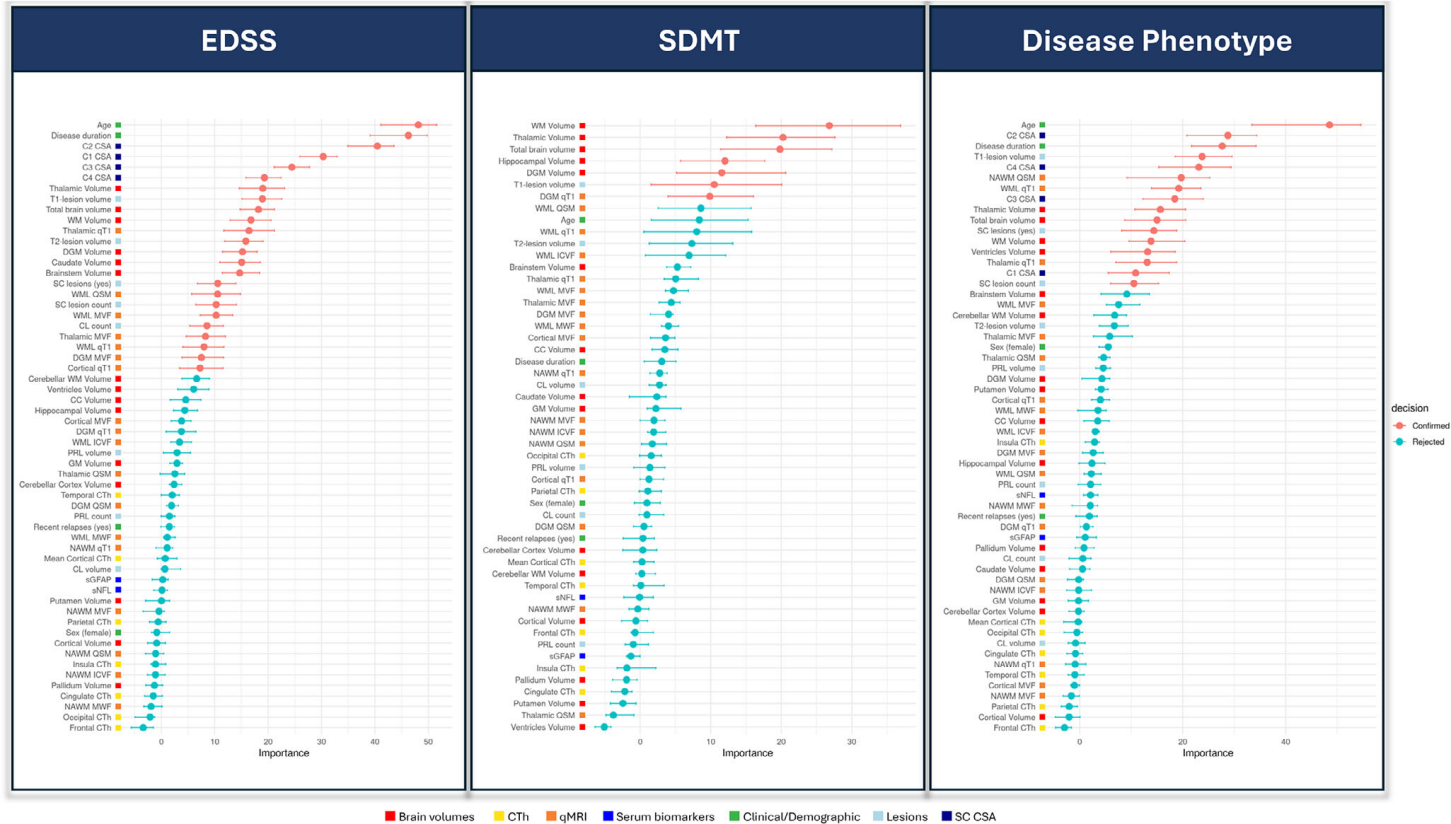
Selected predictors of the EDSS, SDMT, and disease phenotype in Cohort 1. Abbreviations: CC, corpus callosum; CL, cortical lesion; CSA, cross‐sectional area; CTh, cortical thickness; DGM, deep gray matter; GM, gray matter; ICVF, intracellular volume fraction; MVF, myelin volume fraction from magnetization transfer saturation; MWF, myelin water fraction; NAWM, normal‐appearing white matter; PRL, paramagnetic rim lesions; qMRI, quantitative MRI; QSM, quantitative susceptibility mapping; qT1, quantitative T1; RMS, relapsing multiple sclerosis; SC, spinal cord; sGFAP, serum glial fibrillary acidic protein; sNfL, serum neurofilament light chain; WM, white matter; WML, white matter lesions.

Twenty‐four variables were identified by the Boruta model as significant predictors of the EDSS score, with age and disease duration being the strongest. Among biomarkers, spinal cord volumetric measurements emerged as the most critical, with C2, C1, C3, and C4 CSA contributing the most to the prediction model. Additional selected biomarkers included brain volumetric measurements, T1‐ and T2‐WML burden, spinal cord and CL burden, and qMRI metrics in both lesions and normal‐appearing GM (Figure 3). The model incorporating these predictors achieved a mean R^2^ of 0.55.

The most relevant biomarkers for distinguishing patients with an EDSS score ≥3.0 (n = 69) were thalamic qT1 and C2 CSA (model mean AUC = 0.91), while for differentiating those with an EDSS score ≥6.0 (n = 24), the key biomarkers were C2 and C1 CSA (model mean AUC = 0.93) (Figure S1).

#### 3.1.2 SDMT Prediction

Seven variables emerged as significant predictors of the SDMT z‐score. These included, in order of importance, brain volumetric measurements (WM, thalamic, total brain, hippocampal, and DGM volumes), T1‐WML burden, and DGM qT1 (Figure 3). The model incorporating these predictors achieved a mean R^2^ of 0.31. In the sensitivity analysis, similar results, confirming the same volumetric variables, were observed when SDMT z‐scores were recalculated based on the HC population rather than normative data (mean R^2^ = 0.25) (Figure S2).

#### 3.1.3 Prediction of the Disease Phenotype

Sixteen variables significantly contributed to differentiating between PMS and RRMS, with age being the strongest predictor, followed by C2 CSA. Other selected biomarkers included T1‐WML burden, C4, C3, and C1 CSA, qMRI metrics in both WMLs and normal‐appearing tissue, and spinal cord lesion burden (Figure 3). The model incorporating these predictors achieved a mean AUC of 0.92. When EDSS was included as an additional predictor, it emerged as the strongest determinant, while the other selected biomarkers were largely consistent with the main analysis (mean AUC = 1.00) (Figure S3).

#### 3.1.4 Prediction of Time to PIRA

During the follow‐up period, 28 pwMS experienced PIRA and 26 pwMS experienced PIRMA. Among them, 16 patients with PIRA and 15 patients with PIRMA had an RRMS course. Only one patient experienced relapse‐associated worsening.

**FIGURE 4:**
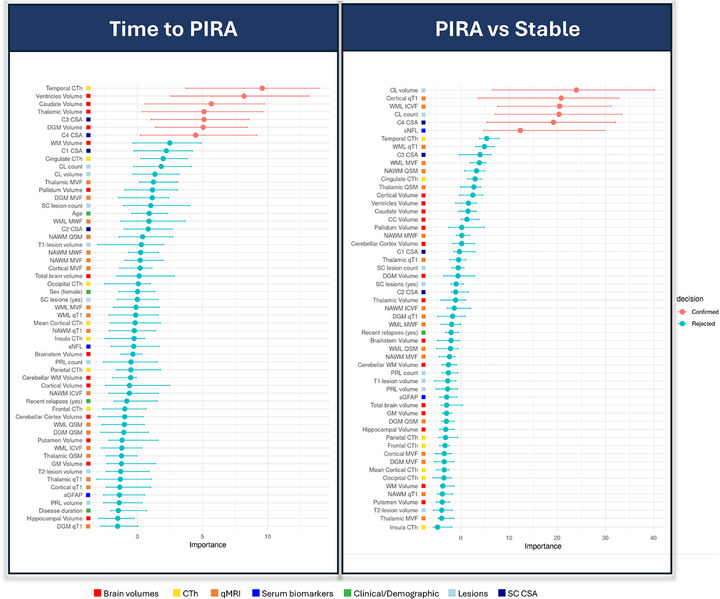
Selected predictors of PIRA in Cohort 1. Abbreviations: CC, corpus callosum; CL, cortical lesion; CSA, cross‐sectional area; CTh, cortical thickness; DGM, deep gray matter; GM, gray matter; ICVF, intracellular volume fraction; MVF, myelin volume fraction from magnetization transfer saturation; MWF, myelin water fraction; NAWM, normal‐appearing white matter; PIRA, progression independent of relapse activity; PRL, paramagnetic rim lesions; qMRI, quantitative MRI; QSM, quantitative susceptibility mapping; qT1, quantitative T1; RMS, relapsing multiple sclerosis; SC, spinal cord; sGFAP, serum glial fibrillary acidic protein; sNfL, serum neurofilament light chain; WM, white matter; WML, white matter lesions.

Seven variables emerged as significant predictors of time to PIRA. These included, in order of importance, CTh in the temporal lobe, volumetric measurements of the ventricles, caudate, DGM and thalamus, and C3 and C4 CSA (Figure 4). The selected variables yielded a mean C‐index of 0.67. Results remained consistent when including EDSS as an additional predictor (mean C‐index = 0.70).

When predicting PIRMA events, five variables were selected: CTh in the temporal lobe, DGM volume, C1 CSA, ventricular volume, and thalamic volume (mean C‐index = 0.70) (Figure S4).

In the RRMS subgroup, CTh in the temporal lobe was the only significant predictor of both PIRA (mean C‐index = 0.64) and PIRMA (mean C‐index = 0.69) (Figure S5).

In the propensity score‐matched analysis, six variables were identified as discriminators between patients with and without PIRA, including measures of cortical damage (CL volume, CL count, and cortical qT1), neuroaxonal density within WMLs (ICVF), spinal cord C4 CSA, and sNfL levels (model mean AUC = 0.80) (Figure 4). Group characteristics before and after propensity score‐matching are reported in Table S5.

In SHAP dependence plots aimed at exploring the relationship between individual predictors and model‐predicted PIRA risk, lower DGM volumes, reduced cervical CSA, and increased ventricular volume were associated with higher model‐predicted PIRA risk. Temporal CTh displayed a non‐linear association, with lower thickness corresponding to higher predicted PIRA risk (Figure S6). The ridge Cox model confirmed the direction of associations observed in the SHAP analyses (Figure S7).

### 3.2 Validation Cohort

A total of 279 pwMS were included in Cohort 2; key characteristics are reported in Table 1. The median follow‐up time was 7.3 years (IQR: 6.7; 7.9).

**FIGURE 5:**
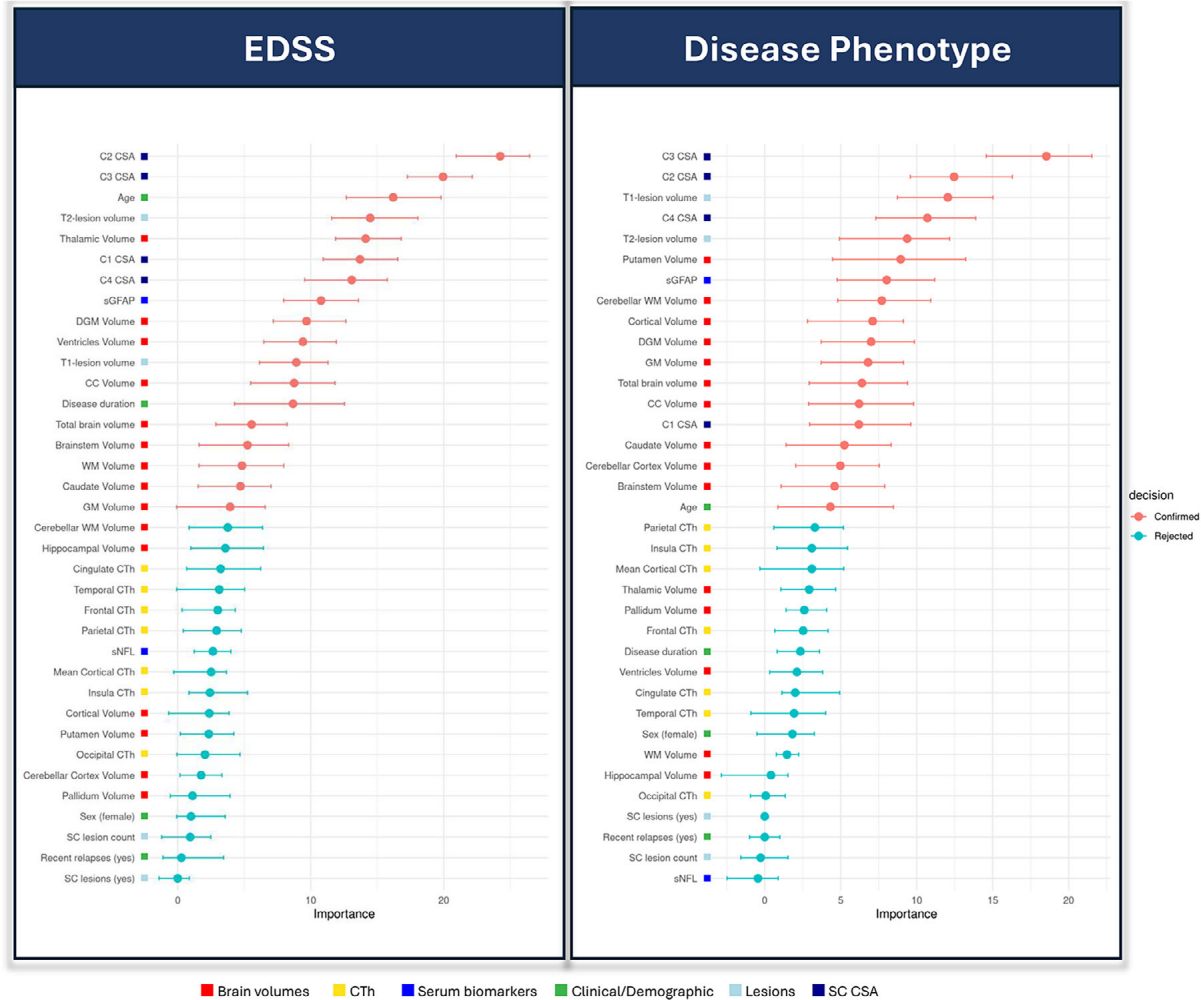
Selected predictors of the EDSS and disease phenotype in Cohort 2. Abbreviations: CC, corpus callosum; CI, confidence interval; CL, cortical lesion; CSA, cross‐sectional area; CTh, cortical thickness; DGM, deep gray matter; GM, gray matter; SC, spinal cord; sGFAP, serum glial fibrillary acidic protein; sNfL, serum neurofilament light chain; WM, white matter; WML, white matter lesions.

Eighteen variables were identified as significant predictors of the EDSS score. As in Cohort 1, spinal cord volumetric measurements – particularly CSA at all cervical levels – were among the strongest contributors. Additional selected biomarkers included T2‐ and T1‐WML volume, sGFAP levels, and several brain volumetric measures, with thalamic volume again emerging as the most relevant (Figure 5). The model achieved a mean R^2^ of 0.34. Models discriminating pwMS based on EDSS cut‐offs of ≥3.0 and ≥6.0 achieved mean AUCs of 0.79 and 0.92, respectively (Figure S8).

Eighteen variables also significantly contributed to distinguishing between RRMS and PMS. As in Cohort 1, spinal cord volumetric measures were the most important, alongside T1‐ and T2‐WML volume, sGFAP values, and multiple brain volumetric metrics (model mean AUC = 0.87) (Figure 5).

**FIGURE 6:**
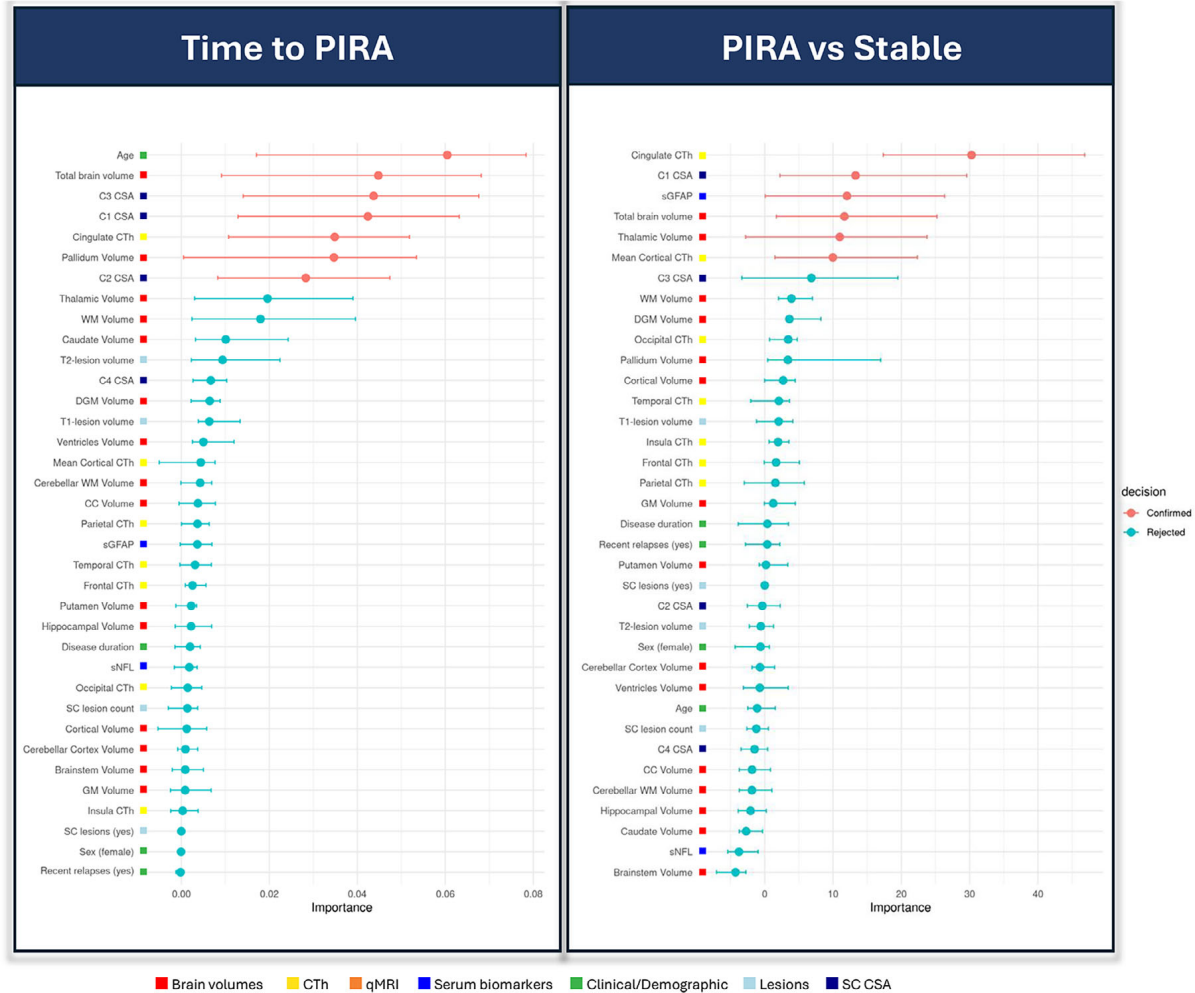
Selected predictors of PIRA in Cohort 2. Abbreviations: CC, corpus callosum; CI, confidence interval; CL, cortical lesion; CSA, cross‐sectional area; CTh, cortical thickness; DGM, deep gray matter; GM, gray matter; SC, spinal cord; sGFAP, serum glial fibrillary acidic protein; sNfL, serum neurofilament light chain; WM, white matter; WML, white matter lesions.

During follow‐up, 79 pwMS experienced PIRA. Seven variables were selected as predictors of time to PIRA. These included CSA at the C1, C2, and C3 levels, total brain volume, pallidum volume, and cingulate CTh (model mean C‐index: 0.70) (Figure 6).

In the RRMS subgroup, the top predictors of time to PIRA, in order of importance, were C3 CSA, pallidum volume, thalamic volume, mean CTh, and caudate volume (model mean C‐index: 0.76) (Figure S9).

Fifty‐six out of the 79 pwMS with PIRA fulfilled criteria for PIRMA. The same five variables – C3 CSA, pallidum volume, thalamic volume, mean CTh, and caudate volume – were selected as predictors of time to PIRMA (model mean C‐index: 0.81) (Figure S10).

In the propensity score‐matched analysis, six variables emerged as discriminators between patients with and without PIRA. The most relevant was cingulate CTh, followed by C1 CSA, sGFAP levels, total brain volume, thalamic volume, and mean CTh (model mean AUC = 0.67) (Figure 6).

Conditional Boruta in both cohorts produced findings that were overall comparable with the main analyses, although with a more parsimonious set of selected predictors (Figures S11–S14).

### 3.3 Derivation and Validation of a Clinically Accessible PIRA Risk Score

To explore the feasibility of deriving a clinically applicable risk score for PIRA based on widely available MRI biomarkers, we developed a parsimonious prognostic score using ridge regression. From the subset of predictors identified by the Boruta analysis in Cohort 2, we selected variables with broad clinical availability, resulting in a model including age, C1 CSA, normalized total brain volume, and normalized thalamic volume. This score was derived in Cohort 2. In this cohort, the risk score was strongly associated with time to PIRA (hazard ratio [HR] 3.30, 95% CI 1.99–5.62; *p* <0.0001) and achieved a C‐index of 0.64. Time‐dependent AUCs calculated between 2 and 7 years were all statistically significant, ranging from 0.81 at 3 years to 0.61 at 7 years (Table S6).

When applied to Cohort 1, the risk score showed consistent prognostic performance, with an HR of 3.60 (95% CI 1.57–8.25; *p* = 0.002) and a C‐index of 0.67 (Figure S15). Time‐dependent AUCs between 2 and 4 years were all significant, with values ranging from 0.68 at 2 years to 0.73 at 3 years (Table S7).

## 4 Discussion

In this prospective cross‐sectional and longitudinal study, we assessed the relative importance of conventional and advanced MRI biomarkers of brain and spinal cord damage, alongside serum biomarkers, in predicting key clinical outcomes in pwMS. A machine learning approach was employed to identify the variables most significantly contributing to clinical outcomes and to rank their importance. Our analysis suggested several predictors of neurological disability, cognitive impairment, as well as future disability progression, including spinal cord and brain volumetric metrics, qMRI parameters, and measures of lesion burden. Among the evaluated biomarkers, spinal cord atrophy emerged as a relevant determinant of disability severity and a distinguishing feature of patients with progressive disease. Cortical damage, encompassing both focal lesion burden and diffuse neurodegeneration, contributed to predicting the risk of PIRA. Notably, PIRA was also linked to markers of spinal cord and deep gray matter damage, in addition to sNfL levels. The reproducibility of these findings was broadly consistent in an independent validation cohort of 279 pwMS with a longer follow‐up. In this second cohort, spinal cord CSA measurements at all cervical levels again emerged as top predictors of EDSS, reinforcing their central role in determining physical disability. Thalamic volume, lesion burden, and sGFAP levels also contributed meaningfully to model performance. Consistent predictors of time to PIRA included spinal cord atrophy, deep gray matter volume, and cortical thickness, particularly in the cingulate cortex. These findings support the relevance of the selected biomarkers in capturing both cross‐sectional disability and future disease progression.

Pathological changes in the spinal cord are a hallmark of MS, often manifesting early in the disease course and becoming particularly pronounced in PMS [8]. These changes include both focal demyelinating lesions and diffuse degeneration leading to atrophy. Spinal cord lesions are associated with an increased risk of disease progression [35], while spinal cord atrophy has been consistently linked to clinical disability in several studies [8, 36]. Atrophy reflects a complex interplay of microstructural changes, including demyelination, neuroaxonal loss, and gliosis [37], and progresses partially independently of both spinal cord lesions and brain pathology [38]. Importantly, spinal cord atrophy has been shown to predict future clinical worsening [39], including PIRA [23, 40]. In this study, spinal cord atrophy emerged as the most significant marker of disability severity, with CSA at the four rostral cervical cord levels identified as primary predictors of the EDSS score. Among these, C2 CSA was the most informative biomarker for distinguishing PMS from RRMS. Furthermore, spinal cord CSA predicted disease progression due to PIRA. Notably, the influence of spinal cord CSA on disability severity and progression was independent of spinal cord lesion burden, which played a comparatively minor role. Collectively, these findings underscore spinal cord degeneration as a key determinant of MS severity. The validation cohort further reinforced these conclusions, with CSA at C1–C3 levels among the most important predictors of both overall disability and time to PIRA. Notably, C3 CSA was the strongest predictor of progression in the RRMS subgroup, supporting its robustness as a marker across disease phenotypes. Remarkably, these findings are noteworthy given that spinal cord CSA was derived directly from brain MRI scans. Although coverage was limited to the upper cervical cord and no specific spinal cord imaging protocol was available, the results indicate that meaningful and clinically relevant information can be obtained from cervical spinal cord segments that are commonly included in standard brain MRI examinations.

Measures of cortical damage also emerged as crucial predictors of clinical outcomes. Cortical degeneration, encompassing both focal lesions and diffuse atrophy reflected by volume loss and thinning, is a critical yet often underappreciated manifestation of MS pathology. Focal CLs, long overlooked due to imaging limitations, are now recognized as significant contributors to disease progression, linked to inflammation, demyelination, and neuronal loss [2, 41]. Cortical atrophy correlates with cognitive decline and physical disability, progressing steadily and most prominently in progressive disease forms [8, 42]. This degeneration arises from a complex interplay of primary neurodegenerative processes and secondary effects of white matter damage [43], occurring in non‐random clinically‐relevant anatomical patterns [44]. Advances in imaging have enhanced the detection of cortical abnormalities, including both lesions and atrophy, providing valuable insights into their role as predictors of long‐term disability and their potential as therapeutic targets [7]. In our study, while cortical damage was significantly associated with disease severity, its contribution was relatively lower than other MRI‐based metrics. However, cortical damage was pivotal in predicting PIRA. Remarkably, CTh in the temporal lobe emerged as the strongest predictor of time to PIRA in Cohort 1, even in sensitivity analyses restricted to PIRMA events, and restricted to PIRA events that occurred in patients with a classical RRMS disease course. Cerebral cortex has previously been shown to exhibit accelerated thinning in patients experiencing PIRA as compared to stable patients [42]. Interestingly, our findings also suggest a potential connection with results from a large longitudinal study, which identified accelerated GM atrophy in the temporal lobe as the only volumetric measurement distinguishing patients with secondary progressive MS from those with RRMS [45]. In line with these findings, the validation cohort confirmed the predictive value of CTh – particularly cingulate and mean CTh – for PIRA and PIRMA.

Propensity score‐matched analysis in Cohort 1 further underscored the importance of focal cortical degeneration in explaining disability accumulation due to PIRA. CLs showed the highest importance in distinguishing patients with PIRA from stable patients, with both CL volume and count being significant predictors. Interestingly, a previous study has likewise linked CL burden to the risk of conversion to secondary progressive MS [2]. Additionally, mean qT1 values in the normal‐appearing cerebral cortex significantly contributed to the classification, highlighting their relevance as a measure sensitive to tissue micro‐ and macrostructural integrity, influenced by myelin, water, and iron content [6].

As expected, the biomarkers most associated with performance on the SDMT differed from those predicting physical disability. Subcortical volumetric measurements – including white matter, thalamus, hippocampus, and deep GM – emerged as the most relevant predictors. These findings align with prior studies emphasizing the importance of subcortical structures in explaining cognitive performance, particularly attention and information processing speed in pwMS [46, 47]. The relevance of subcortical degeneration was further supported by the importance of qT1 values in the deep GM as a measure of tissue degeneration and loss of structural integrity.

The results of this study highlighted the complementary roles of brain and spinal cord volumetric measurements alongside measures of focal lesion burden. While CL load and T1‐hypointense WMLs were significant predictors of the clinical outcomes under consideration, PRLs showed predictive value in univariate analyses but did not provide additional information in multivariate models. This finding may reflect the enhanced insights offered by advanced qMRI metrics, which capture WML microstructural changes with greater sensitivity. Alternatively, it could be partially attributable to the limited statistical power of the study, underscoring the need for further investigation with larger cohorts. Notably, qMRI metrics of normal‐appearing tissue also significantly contributed to predicting disease severity and progression. Additionally, sNfL and sGFAP levels were useful in predicting PIRA. Elevated sNfL levels, a biomarker of neuroaxonal injury, are valuable for detecting acute and chronic neuronal damage. Existing literature has also shown the potential value of sNfL in predicting PIRA [10, 16, 48]. Given its increasing availability in clinical settings, sNfL is a promising biomarker; however, previous studies have highlighted sGFAP as a complementary marker that may offer greater specificity for smoldering disease activity and lower sensitivity to acute inflammation [10, 49]. Indeed, in the validation cohort, sGFAP emerged as a significant predictor not only of disability severity and disease phenotype but also of PIRA occurrence.

In this study, we identified several predictors of PIRA, predominantly reflecting cortical degeneration, cervical spinal cord atrophy, and deep gray matter damage. These predictors yielded overall moderate performance in time‐to‐event models, with C‐indices ranging from 0.64 to 0.81 across analyses. This finding is consistent with the known complexity of predicting an outcome such as PIRA, which is intrinsically challenging. Silent progression is influenced by multiple, partially overlapping biological and clinical mechanisms. In addition, its identification and the determination of its onset are technically challenging due to reliance on EDSS‐based definitions, measurement noise, variability in follow‐up intervals, and the influence of confounding factors such as aging and comorbidities. Together, these factors likely contribute to the moderate discriminative performance observed, which is nonetheless comparable to – or in some cases higher than – previous investigations [50, 51, 52, 53, 54]. Importantly, a prediction score derived from a limited set of clinically accessible biomarkers achieved consistent performance and was corroborated across the two cohorts, suggesting that meaningful risk stratification is achievable to a certain extent. Such an approach may be particularly valuable for research applications and may also support clinical monitoring by facilitating the identification of individuals at potentially higher risk of silent progression.

A key strength of this study is the simultaneous evaluation of a diverse array of MRI and serum biomarkers, providing a comprehensive assessment of their relative contributions to clinical outcomes. The machine learning approach employed for feature selection reduced noise and overfitting while maintaining model interpretability, enabling the handling of complex, non‐linear relationships. Models based on the predictors selected by Boruta showed high performance for disability classification and moderate performance for PIRA prediction, in line with the inherent complexity of this outcome. Importantly, results from the second cohort corroborated these overall patterns.

Nevertheless, the study has some limitations. First, PIRA was assessed exclusively through EDSS, which may underestimate subtle progression with still significant clinical impact. Second, the absence of spinal cord regional damage assessment is a limitation, especially considering recent studies showing that spinal cord GM atrophy outperforms whole CSA in explaining disability [33, 55]. Third, although the inclusion of a validation cohort strengthens the robustness of our findings, several differences between cohorts should be acknowledged. The cohorts differed in follow‐up duration – an important factor when studying outcomes such as PIRA – and in baseline clinical characteristics, with Cohort 1 including a higher proportion of progressive MS and higher baseline EDSS. These differences may partly explain discrepancies in the specific biomarkers selected across cohorts. In addition, fewer biomarkers were available in Cohort 2, and the absence of a matched healthy control cohort precluded adjustment of imaging biomarkers for physiological confounders using deviation‐from‐normal approaches. Despite these differences, we deliberately included the second cohort to enhance generalizability, acknowledging that cohorts with an availability of advanced MRI metrics as extensive as Cohort 1 are exceptionally rare. Importantly, both cohorts consistently highlighted the relevance of cortical and spinal cord damage, and predictors of PIRA identified in Cohort 2 generalized well when applied to Cohort 1 for prognostication, supporting the robustness of the identified biomarkers across heterogeneous populations. Fourth, the disproportionate inclusion of a broad set of MRI‐derived biomarkers compared to only two serum biomarkers (sNfL and sGFAP) may have introduced a bias toward the selection of MRI features in the variable importance ranking. Although serum biomarkers were associated with PIRA, the study was not powered to determine whether their addition to quantitative MRI metrics yields a statistically robust improvement in predictive performance, and further studies specifically designed to address this question will be required. Finally, despite the inclusion of two independent cohorts and a relatively long follow‐up, it should be acknowledged that PIRA occurred only in a subset of patients, representing a common challenge in PIRA research. Consequently, the absolute number of PIRA events was limited, which may affect the generalizability of the findings. Nevertheless, the consistency of results across cohorts supports the robustness of the observed associations, while further confirmation in larger populations and additional settings will be important to refine and validate these predictive approaches.

Despite these limitations, the findings are of potential clinical relevance. They highlight the crucial role of spinal cord and cortical degeneration as prognostic markers in MS, underscoring the potential value of their inclusion in clinical evaluations to bridge the gap between conventional MRI biomarkers and measures of disease severity and progression. These insights potentially pave the way for more refined and targeted approaches to understanding and managing MS.

## Funding

No specific funding was received for this work.

## Conflicts of Interest

No specific conflict of interest related to this work.

## Supporting information




**Supporting File**: advs73801‐sup‐0001‐SuppMat.docx.

## Data Availability

The data that support the findings of this study are available from the corresponding author upon reasonable request.

## References

[1] F. Barkhof , “The Clinico‐radiological Paradox in Multiple Sclerosis Revisited,” Current Opinion in Neurology 15, no. 3 (2002): 239–245, 10.1097/00019052-200206000-00003. 12045719

[2] A. Scalfari , C. Romualdi , R. S. Nicholas , et al., “The Cortical Damage, Early Relapses, and Onset of the Progressive Phase in Multiple Sclerosis,” Neurology 90, no. 24 (2018): e2099–e2106, 10.1212/WNL.0000000000005685. 29769373

[3] M. Calabrese , M. Filippi , and P. Gallo , “Cortical Lesions in Multiple Sclerosis,” Nature Reviews Neurology 6, no. 8 (2010): 438–444, 10.1038/nrneurol.2010.93. 20625376

[4] F. Bagnato , P. Sati , C. C. Hemond , et al., “Imaging Chronic Active Lesions in Multiple Sclerosis: A Consensus Statement,” Brain 147, no. 9 (2024): 2913–2933, 10.1093/BRAIN/AWAE013. 38226694 PMC11370808

[5] M. Absinta , P. Sati , F. Masuzzo , et al., “Association of Chronic Active Multiple Sclerosis Lesions with Disability in Vivo,” JAMA Neurology 76, no. 12 (2019): 1474, 10.1001/JAMANEUROL.2019.2399. 31403674 PMC6692692

[6] C. Granziera , J. Wuerfel , F. Barkhof , et al., “Quantitative Magnetic Resonance Imaging towards Clinical Application in Multiple Sclerosis,” Brain 144, no. 5 (2021): 1296–1311, 10.1093/brain/awab029. 33970206 PMC8219362

[7] A. Cagol , C. Tsagkas , and C. Granziera , “Advanced Brain Imaging in Central Nervous System Demyelinating Diseases,” Neuroimaging Clinics 34, no. 3 (2024): 335–357, 10.1016/J.NIC.2024.03.003. 38942520

[8] J. Sastre‐Garriga , D. Pareto , M. Battaglini , et al., “MAGNIMS Consensus Recommendations on the Use of Brain and Spinal Cord Atrophy Measures in Clinical Practice,” Nature Reviews Neurology 16, no. 3 (2020): 171–182, 10.1038/s41582-020-0314-x. 32094485 PMC7054210

[9] F. M. Di , G. L , D. Centonze , et al., “Fluid Biomarkers in Multiple Sclerosis: from Current to Future Applications,” The Lancet Regional Health – Europe 44 (2024): 101009, 10.1016/J.LANEPE.2024.101009. 39444698 PMC11496979

[10] S. Meier , E. A. J. Willemse , S. Schaedelin , et al., “Serum Glial Fibrillary Acidic Protein Compared with Neurofilament Light Chain as a Biomarker for Disease Progression in Multiple Sclerosis,” JAMA Neurology 80 (2023): 287–297, 10.1001/jamaneurol.2022.5250. 36745446 PMC10011932

[11] P. Benkert , A. Maleska Maceski , S. Schaedelin , et al., “Serum Glial Fibrillary Acidic Protein and Neurofilament Light Chain Levels Reflect Different Mechanisms of Disease Progression under B‐Cell Depleting Treatment in Multiple Sclerosis,” Annals of Neurology 97, no. 1 (2025): 104–115, 10.1002/ANA.27096. PMC11683165 39411917

[12] O. Ciccarelli , F. Barkhof , M. Calabrese , et al., “Using the Progression Independent of Relapse Activity Framework to Unveil the Pathobiological Foundations of Multiple Sclerosis,” Neurology 103, no. 1 (2024): 209444, 10.1212/WNL.0000000000209444/SUPPL_FILE/SUPPLEMENTARY_TABLE1.PDF. PMC11226318 38889384

[13] G. Disanto , P. Benkert , J. Lorscheider , et al., “The Swiss Multiple Sclerosis Cohort‐Study (SMSC): A Prospective Swiss Wide Investigation of Key Phases in Disease Evolution and New Treatment Options,” PLoS One 11, no. 3 (2016): 0152347, 10.1371/journal.pone.0152347. PMC4816556 27032105

[14] A. J. Thompson , B. L. Banwell , F. Barkhof , et al., “Diagnosis of Multiple Sclerosis: 2017 Revisions of the McDonald Criteria,” Lancet Neurology 17, no. 2 (2018): 162–173, 10.1016/S1474-4422(17)30470-2. 29275977

[15] L. Kappos , H. Butzkueven , H. Wiendl , et al., “Greater Sensitivity to Multiple Sclerosis Disability Worsening and Progression Events Using a Roving versus a Fixed Reference Value in a Prospective Cohort Study,” Multiple Sclerosis Journal 24, no. 7 (2018): 963–973, 10.1177/1352458517709619. 28554238 PMC6029149

[16] J. Müller , A. Cagol , J. Lorscheider , et al., “Harmonizing Definitions for Progression Independent of Relapse Activity in Multiple Sclerosis: a Systematic Review,” JAMA Neurology 80, no. 11 (2023): 1232–1245, 10.1001/jamaneurol.2023.3331. 37782515

[17] N. Montobbio , L. Carmisciano , A. Signori , et al., “Creating an Automated Tool for a Consistent and Repeatable Evaluation of Disability Progression in Clinical Studies for Multiple Sclerosis,” Multiple Sclerosis Journal 30 (2024): 1185–1192, 10.1177/13524585241243157/SUPPL_FILE/SJ-DOCX-1-MSJ-10.1177_13524585241243157.DOCX. 39143826

[18] A. Smith , Symbol Digit Modalities Test , last modified November 24, 2021, https://www.communicate‐ed.org.uk/assets/downloads/SDMT_Formula_Chart_Communicate‐ed_2.pdf.

[19] R. Rahmanzadeh , M. Weigel , P. J. Lu , et al., “A Comparative Assessment of Myelin‐Sensitive Measures in Multiple Sclerosis Patients and Healthy Subjects,” Neuroimage Clin 36 (2022): 103177, 10.1016/j.nicl.2022.103177. 36067611 PMC9468574

[20] F. La Rosa , A. Abdulkadir , M. J. Fartaria , et al., “Multiple Sclerosis Cortical and WM Lesion Segmentation at 3T MRI: A Deep Learning Method Based on FLAIR and MP2RAGE,” Neuroimage Clincal 27 (2020): 102335, 10.1016/j.nicl.2020.102335. PMC7358270 32663798

[21] A. Cagol , R. Cortese , M. Barakovic , et al., “Diagnostic Performance of Cortical Lesions and the Central Vein Sign in Multiple Sclerosis,” JAMA Neurology 81, no. 2 (2024): 143–153, 10.1001/JAMANEUROL.2023.4737. 38079177 PMC10714285

[22] S. Cerri , O. Puonti , D. S. Meier , et al., “A Contrast‐ADAPTIVE Method for Simultaneous Whole‐brain and Lesion Segmentation in Multiple Sclerosis,” Neuroimage 225 (2021): 117471, 10.1016/j.neuroimage.2020.117471. 33099007 PMC7856304

[23] A. Cagol , P. Benkert , L. Melie‐Garcia , et al., “Association of Spinal Cord Atrophy and Brain Paramagnetic Rim Lesions with Progression Independent of Relapse Activity in People with MS,” Neurology 102, no. 1 (2024): 207768, 10.1212/WNL.0000000000207768. PMC10834139 38165377

[24] O. Puonti , J. E. Iglesias , and K. Van Leemput , “Fast and Sequence‐Adaptive Whole‐brain Segmentation Using Parametric Bayesian Modeling,” Neuroimage 143 (2016): 235–249, 10.1016/j.neuroimage.2016.09.011. 27612647 PMC8117726

[25] B. De Leener , S. Lévy , S. M. Dupont , et al., “SCT: Spinal Cord Toolbox, an Open‐Source Software for Processing Spinal Cord MRI Data,” Neuroimage 145 (2017): 24–43, 10.1016/j.neuroimage.2016.10.009. 27720818

[26] J. P. Marques , T. Kober , G. Krueger , W. van der Zwaag , P. F. Van de Moortele , and R. Gruetter , “MP2RAGE, a Self Bias‐Field Corrected Sequence for Improved Segmentation and T1‐Mapping at High Field,” Neuroimage 49, no. 2 (2010): 1271–1281, 10.1016/j.neuroimage.2009.10.002. 19819338

[27] T. D. Nguyen , K. Deh , E. Monohan , et al., “Feasibility and Reproducibility of Whole Brain Myelin Water Mapping in 4 Minutes Using Fast Acquisition with Spiral Trajectory and Adiabatic T2prep (FAST‐T2) at 3T,” Magnetic Resonance in Medicine 76, no. 2 (2016): 456–465, 10.1002/mrm.25877. 26331978 PMC5486993

[28] H. Zhang , T. Schneider , C. A. Wheeler‐Kingshott , and D. C. Alexander , “NODDI: Practical In Vivo Neurite Orientation Dispersion and Density Imaging of the human Brain,” Neuroimage 61, no. 4 (2012): 1000–1016, 10.1016/j.neuroimage.2012.03.072. 22484410

[29] T. Liu , W. Xu , P. Spincemaille , A. S. Avestimehr , and Y. Wang , “Accuracy of the Morphology Enabled Dipole Inversion (MEDI) Algorithm for Quantitative Susceptibility Mapping in MRI,” 31, no. 3 (2012): 816–824, 10.1109/TMI.2011.2182523. PMC3613569 22231170

[30] A. R. Todea , L. Melie‐Garcia , M. Barakovic , et al., “A Multicenter Longitudinal MRI Study Assessing LeMan‐PV Software Accuracy in the Detection of White Matter Lesions in Multiple Sclerosis Patients,” Journal of Magnetic Resonance Imaging 58, no. 3 (2023): 864–876, 10.1002/jmri.28618. 36708267

[31] P. Benkert , S. Meier , S. Schaedelin , et al., “Serum Neurofilament Light Chain for Individual Prognostication of Disease Activity in People with Multiple Sclerosis: A Retrospective Modelling and Validation Study,” Lancet Neurology 21, no. 3 (2022): 246–257, 10.1016/S1474-4422(22)00009-6. 35182510

[32] M. B. Kursa and W. R. Rudnicki , “Feature Selection with the Boruta Package,” Journal of Statistical Software 36, no. 11 (2010): 1–13, 10.18637/JSS.V036.I11.

[33] T. Morozumi , P. Preziosa , A. Meani , et al., “Brain and Cervical Spinal Cord MRI Correlates of Sensorimotor Impairment in Patients with Multiple Sclerosis,” Multiple Sclerosis Journal 30 (2024): 1004–1015, 10.1177/13524585241260145. 38912804

[34] A. Eshaghi , V. Wottschel , R. Cortese , et al., “Gray Matter MRI Differentiates Neuromyelitis Optica from Multiple Sclerosis Using Random Forest,” Neurology 87, no. 23 (2016): 2463–2470, 10.1212/WNL.0000000000003395. 27807185 PMC5177679

[35] W. J. Brownlee , D. R. Altmann , F. Prados , et al., “Early Imaging Predictors of Long‐Term Outcomes in Relapse‐Onset Multiple Sclerosis,” Brain 142, no. 8 (2019): 2276–2287, 10.1093/BRAIN/AWZ156. 31342055

[36] C. Casserly , E. E. Seyman , P. Alcaide‐Leon , et al., “Spinal Cord Atrophy in Multiple Sclerosis: a Systematic Review and Meta‐Analysis,” Journal of Neuroimaging 28, no. 6 (2018): 556–586, https://pubmed.ncbi.nlm.nih.gov/30102003/. 30102003 10.1111/jon.12553

[37] J. C. J. Bot , E. L. A. Blezer , W. Kamphorst , et al., “The Spinal Cord in Multiple Sclerosis: Relationship of High‐spatial‐resolution Quantitative MR Imaging Findings to Histopathologic Results,” Radiology 233, no. 2 (2004): 531–540, 10.1148/RADIOL.2332031572. 15385682

[38] N. Evangelou , G. C. DeLuca , T. Owens , and M. M. Esiri , “Pathological Study of Spinal Cord Atrophy in Multiple Sclerosis Suggests Limited Role of Local Lesions,” Brain 128, no. 1 (2005): 29–34, 10.1093/BRAIN/AWH323. 15548559

[39] M. A. Rocca , P. Valsasina , A. Meani , et al., “Spinal Cord Lesions and Brain Grey Matter Atrophy Independently Predict Clinical Worsening in Definite Multiple Sclerosis: A 5‐year, Multicentre Study,” Journal of Neurology, Neurosurgery & Psychiatry 94, no. 1 (2023): 10–18, 10.1136/JNNP-2022-329854. 36171105

[40] A. Bischof , N. Papinutto , A. Keshavan , et al., “Spinal Cord Atrophy Predicts Progressive Disease in Relapsing Multiple Sclerosis,” Annals of Neurology 91, no. 2 (2022): 268–281, 10.1002/ana.26281. 34878197 PMC8916838

[41] M. Calabrese , M. A. Rocca , M. Atzori , et al., “A 3‐year Magnetic Resonance Imaging Study of Cortical Lesions in Relapse‐onset Multiple Sclerosis,” Annals of Neurology 67, no. 3 (2010): 376–383, 10.1002/ANA.21906. 20373349

[42] A. Cagol , S. Schaedelin , M. Barakovic , et al., “Association of Brain Atrophy with Disease Progression Independent of Relapse Activity in Patients with Relapsing Multiple Sclerosis,” JAMA Neurology 79, no. 7 (2022): 682–692, 10.1001/jamaneurol.2022.1025. 35575778 PMC9112138

[43] C. A. Treaba , E. Herranz , V. T. Barletta , et al., “The Relevance of Multiple Sclerosis Cortical Lesions on Cortical Thinning and Their Clinical Impact as Assessed by 7.0‐T MRI,” J Neurol 268, no. 7 (2021): 2473–2481, 10.1007/S00415-021-10400-4. 33523256

[44] M. D. Steenwijk , J. J. G. Geurts , M. Daams , et al., “Cortical Atrophy Patterns in Multiple Sclerosis Are Non‐Random and Clinically Relevant,” Brain 139, no. 1 (2016): 115–126, 10.1093/brain/awv337. 26637488

[45] A. Eshaghi , F. Prados , W. J. Brownlee , et al., “Deep Gray Matter Volume Loss Drives Disability Worsening in Multiple Sclerosis,” Ann Neurol 83, no. 2 (2018): 210–222, 10.1002/ana.25145. 29331092 PMC5838522

[46] A. Bisecco , S. Stamenova , G. Caiazzo , et al., “Attention and Processing Speed Performance in Multiple Sclerosis Is Mostly Related to Thalamic Volume,” Brain Imaging and Behavior 12, no. 1 (2018): 20–28, 10.1007/S11682-016-9667-6/TABLES/5. 28083844

[47] K. A. Koenig , K. E. Sakaie , M. J. Lowe , et al., “Hippocampal Volume Is Related to Cognitive Decline and Fornicial Diffusion Measures in Multiple Sclerosis,” Magnetic Resonance Imaging 32, no. 4 (2013): 354, 10.1016/J.MRI.2013.12.012. 24512796 PMC4025957

[48] A. Abdelhak , P. Benkert , S. Schaedelin , et al., “Neurofilament Light Chain Elevation and Disability Progression in Multiple Sclerosis,” JAMA Neurology 80, no. 12 (2023): 1317–1325, 10.1001/JAMANEUROL.2023.3997. 37930670 PMC10628837

[49] P. Benkert , A. Maleska Maceski , S. Schaedelin , et al., “Serum Glial Fibrillary Acidic Protein and Neurofilament Light Chain Levels Reflect Different Mechanisms of Disease Progression under B‐Cell Depleting Treatment in Multiple Sclerosis,” Annals of Neurology (2024): 104–115, 10.1002/ANA.27096. 39411917 PMC11683165

[50] L. Coll , D. Pareto , F. Aparicio‐Serrano , et al., “Deep Learning to Predict Progression Independent of Relapse Activity at a First Demyelinating Event,” Brain Communications 7, no. 4 (2025): fcaf243, 10.1093/BRAINCOMMS/FCAF243. 40620473 PMC12226453

[51] M. Lauerer , T. Wiltgen , C. Brückner , et al., “Predictors of Early Disability Accumulation in Newly Diagnosed Multiple Sclerosis: Clinical, Imaging and Cerebrospinal Fluid Measures,” Journal of Neurology, Neurosurgery & Psychiatry 96, no. 9 (2025): 900–907, 10.1136/JNNP-2024-335037. 39961655 PMC12418557

[52] E. Monreal , J. I. Fernández‐Velasco , M. I. García‐Sánchez , et al., “Association of Serum Neurofilament Light Chain Levels at Disease Onset with Disability Worsening in Patients with a First Demyelinating Multiple Sclerosis Event Not Treated with High‐Efficacy Drugs,” JAMA Neurology 80, no. 4 (2023): 397–403, 10.1001/JAMANEUROL.2023.0010. 36848127 PMC9972238

[53] V. Poretto , W. Endrizzi , M. Betti , et al., “Machine Learning Analysis Applied to Prediction of Early Progression Independent of Relapse Activity in Multiple Sclerosis Patients,” European Journal of Neurology 32, no. 12 (2025): 70417, 10.1111/ENE.70417;SUBPAGE:STRING:FULL. PMC12661373 41312659

[54] D. Marastoni , E. Colato , M. Foschi , et al., “Intrathecal Inflammatory Profile and Gray Matter Damage Predict Progression Independent of Relapse Activity in Early Multiple Sclerosis,” Neurology(R) Neuroimmunology & Neuroinflammation 12, no. 4 (2025): 200399, 10.1212/NXI.0000000000200399. PMC12056761 40311103

[55] C. Tsagkas , A. Huck‐Horvath , A. Cagol , et al., “Anterior Horn Atrophy in the Cervical Spinal Cord: a New Biomarker in Progressive Multiple Sclerosis,” Multiple Sclerosis Journal 29, no. 6 (2023): 702–718, 10.1177/13524585221139152. 36550626

