# Supplementary material for: Assessing the Relative Importance of Imaging and Serum Biomarkers in Capturing Disability, Cognitive Impairment, and Clinical Progression in Multiple Sclerosis
Source: Adv Sci (Weinh). 2026 Jan 12;13(10):e12946. doi: 10.1002/advs.202512946 (PMC12915075; doi:10.1002/advs.202512946)

**Supplementary material**

**Index**

- **eMethods** (*page 2*)
- **eTable 1**: INsIDER MRI protocol (*page 3*)
- **eTable 2**: SMSC MRI protocol (*page 3*)
- **eTable 3:** List of included predictors in Cohort 1 (*page 4*)
- **eTable 4:** List of included predictors in Cohort 2 (*page 5*)
- **eTable 5**: Characteristics of patients with and without PIRA in Cohort 1, before and after propensity score-matching (*page 6*)
- **eTable 6**: Time-dependent AUCs for PIRA prediction using the ridge-based risk score in Cohort 2 (*page 6*)
- **eTable 7**: Time-dependent AUCs for PIRA prediction using the ridge-based risk score in Cohort 2 (*page 6*)
- **eFigure 1**: Selected predictors of EDSS ≥ 3.0 and EDSS ≥ 6.0 in Cohort 1 (*page 7*)
- **eFigure 2**: Selected predictors of SDMT (z-score based on the scores obtained in healthy controls) in Cohort 1 (*page 8*)
- **eFigure 3**: Selected Predictors of the Disease Phenotype (model including EDSS as an explanatory variable) in Cohort 1 (*page 9*)
- **eFigure 4**: Selected Predictors of PIRMA in Cohort 1 (*page 10*)
- **eFigure 5**: Selected Predictors of PIRA (in patients with RRMS only) in Cohort 1 (*page 11*)
- **eFigure 6**: SHAP dependence plots for predictors of time to PIRA identified by the Boruta model in Cohort 1 (*page 12*)
- **eFigure 7**: Ridge Cox regression model for time to PIRA using Boruta-selected predictors (*page 13*)
- **eFigure 8**: Selected Predictors of EDSS ≥ 3.0 and EDSS ≥ 6.0 in Cohort 2 (*page 14*)
- **eFigure 9**: Selected Predictors of PIRA (in patients with RRMS only) in Cohort 2 (*page 15*)
- **eFigure 10**: Selected Predictors of PIRMA in Cohort 2 (*page 16*)
- **eFigure 11**: Conditional Boruta Models in Cohort 1 – EDSS, SDMT, Disease Phenotype (*page 17*)
- **eFigure 12**: Conditional Boruta Models in Cohort 1 – Time to PIRA, PIRA vs. Stable (*page 18*)
- **eFigure 13**: Conditional Boruta Models in Cohort 2 – EDSS, Disease Phenotype (*page 19*)
- **eFigure 14**: Conditional Boruta Models in Cohort 2 – Time to PIRA, PIRA vs. Stable (*page 20*)
- **eFigure 15**: PIRA-free survival in Cohort 1 stratified by a ridge-based clinical risk score (*page 21*)
- **eFigure 16**: Comparison Between Selected Predictors of EDSS in Cohort 1 and Cohort 2 (*page 22*)
- **eFigure 17**: Comparison Between Selected Predictors of Disease Phenotype in Cohort 1 and Cohort 2 (*page 23*)
- **eFigure 18**: Comparison Between Selected Predictors of PIRA in Cohort 1 and Cohort 2 (*page 24*)

**eMethods**

Cortical lesions

Cortical lesions (CLs) were manually identified and segmented on MP2RAGE images. CLs were defined as hypointense regions relative to the surrounding normal-appearing cortex, spanning ≥3 mm along the main in-plane axis and partially or entirely involving the cortex.^1^ CL detection was performed by two raters, with all cases reviewed by both. Discrepancies were resolved through consensus.

Paramagnetic rim lesions

Paramagnetic rim lesions (PRLs) were defined as discrete FLAIR-hyperintense lesions either completely or partially encircled by a rim of paramagnetic signal, visible in at least one contrast between unwrapped phase and quantitative susceptibility mapping (QSM).^2^ PRL detection was performed by two raters, with all cases reviewed by both. Discrepancies were resolved through consensus. The chronic nature of PRLs was ensured by excluding all lesions that were not present on a 3D-FLAIR image acquired ≥6 months prior. We have previously shown high inter-rater agreement in the identification of PRLs in the same cohort.^2^

Spinal cord lesions

The presence and number of spinal cord lesions within the C1-C4 segment were determined by a single rater on both MP2RAGE and FLAIR images for Cohort 1, and MPRAGE and FLAIR images for Cohort 2.

qMRI

Magnetization transfer saturation (MTsat), intracellular volume fraction (ICVF), myelin water fraction (MWF), and QSM maps were linearly registered to the MP2RAGE space using *FLIRT*,^3^ and the results were visually inspected. The transformation matrices obtained in the process were then used to move the regions of interest (i.e., thalamic lesions and normal-appearing tissue, segmented on the MP2RAGE space) back to the original MTsat, ICVF, MWF, and QSM spaces. For all regions and contrasts of interest, the mean intensity values were extracted in the original space using *fslstats*.^3^

Disease modifying treatment classes

Disease modifying treatment (DMT) classes were defined as follows: platform DMTs (including interferon preparations and glatiramer acetate), oral DMTs (including fingolimod, dimethyl fumarate, teriflunomide, and siponimod), and monoclonal antibody (mAb) DMTs (including natalizumab, rituximab, and ocrelizumab). Patients untreated were classified into a separate group.

PIRA risk score

To derive a parsimonious and clinically accessible risk score for progression independent of relapse activity (PIRA), we fitted a ridge-penalized Cox proportional hazards model in Cohort 2 using predictors selected from the Boruta analysis and chosen based on their wide clinical availability. Prior to model fitting, all continuous predictors were standardized to have mean 0 and standard deviation 1; total brain volume and thalamic volume were normalized by total intracranial volume before standardization. The resulting linear predictor was used to compute an individual-level PIRA risk score according to the following formula:

PIRA_ridge_score = (0.2564 × Age) + (−0.2467 × C1 CSA) + (−0.0069 × Total brain volume) + (−0.1066 × Thalamic volume)

Higher values of the PIRA risk score correspond to higher predicted risk of PIRA. This score was derived in Cohort 2 and subsequently applied without recalibration to Cohort 1 to assess external prognostic performance.

**References**

1. Cagol A, Cortese R, Barakovic M, et al. Diagnostic Performance of Cortical Lesions and the Central Vein Sign in Multiple Sclerosis. *JAMA Neurol*. 2024;81(2):143-153. doi:10.1001/JAMANEUROL.2023.4737

2. Cagol A, Benkert P, Melie-Garcia L, et al. Association of Spinal Cord Atrophy and Brain Paramagnetic Rim Lesions With Progression Independent of Relapse Activity in People With MS. *Neurology*. 2024;102(1). doi:10.1212/WNL.0000000000207768

3. Smith SM, Jenkinson M, Woolrich MW, et al. Advances in functional and structural MR image analysis and implementation as FSL. In: *NeuroImage*. Vol 23. Neuroimage; 2004. doi:10.1016/j.neuroimage.2004.07.051

**eTable 1 – INsIDER MRI protocol**

| **MRI sequence** | **Protocol details** |
| --- | --- |
| 3D fluid attenuated inversion recovery (FLAIR) | TR/TE/TI = 5,000/386/1,800 ms; resolution = 1 × 1 × 1 mm^3^; scan time = 5:40 min |
| 3D magnetization-prepared 2 rapid gradient-echo (MP2RAGE) | TR/TI1/TI2 = 5,000/700/2,500 ms; resolution = 1 × 1 × 1 mm^3^; scan time = 8:20 |
| Multi-shell diffusion | TR/TE/δ/Δ = 4,500/75/19/36 ms; resolution = 1.8 × 1.8 × 1.8 mm3; b-values 0/700/1,000/2,000/3,000 s/mm2 with 12/6/20/45/66 measurements, respectively, per shell; diffusion acquisition with 12 measurements of b-value 0 s/mm2 with reversed phase encoding |
| 3D segmented echo planar imaging (EPI) | TR/TE = 64/35 ms; resolution = 0.67 × 0.67 × 0.67 mm^3^ |
| 3D radiofrequency spoiled gradient-echo acquisitions | Three 3D radiofrequency spoiled gradient-echo acquisitions with predominantly magnetization transfer–weighted (TR/α = 25 ms/5°), proton density–weighted (TR/α = 25 ms/5°), and T1-weighted (TR/α = 11 ms/15°) contrasts, used to obtain magnetization transfer saturation (MTsat) maps (resolution = 1.33 × 1.33 × 1.33 mm^3^); total scan time = 9:45 min |
| Fast acquisition with spiral trajectory and adiabatic T2-prep (FAST-T2) | TR/TE = 7.5/0.5 ms, T2prep times = 0 (T2prep turned off), 7.5, 17.5, 67.5, 147.5, 307.5 ms; resolution = 1.25 × 1.25 × 5 mm^3^; scan time = 4.5 min |

*Abbreviations: TR = repetition time; TE = echo time; TI = inversion time.*

**eTable 2 – SMSC MRI protocol**

| **MRI sequence** | **Protocol details** |
| --- | --- |
| 3D fluid attenuated inversion recovery (FLAIR) | TR/TE/TI = 5,000/280/1,800 ms; resolution = 1 × 1 × 1 mm^3^; flip angle = 120 |
| 3D magnetization-prepared rapid gradient-echo (MPRAGE) | TR/TE/TI = 2,300/3.02/900 ms; resolution = 1 × 1 × 1 mm^3^; flip angle = 9 |
| Scanner: *Skyra_fit, Siemens Healthineers*. Magnetic field strength: 3T. | |

*Abbreviations: TR = repetition time; TE = echo time; TI = inversion time.*

**eTable 3 - List of included predictors in Cohort 1**

| Clinical/demographic variables | Age |
| --- | --- |
|  | Sex |
|  | Disease phenotype (PMS vs RRMS) |
|  | Disease duration |
|  | Recent relapses |
| Brain volumetry | Total brain volume |
|  | GM volume |
|  | WM volume |
|  | DGM volume |
|  | Cortical volume |
|  | Thalamic volume |
|  | Cerebellar cortical volume |
|  | Cerebellar WM volume |
|  | Caudate volume |
|  | Brainstem volume |
|  | CC volume |
|  | Putamen volume |
|  | Hippocampal volume |
|  | Pallidum volume |
|  | Ventricles volume |
| CTh | Mean CTh |
|  | Frontal CTh |
|  | Parietal CTh |
|  | Temporal CTh |
|  | Occipital CTh |
|  | Insular CTh |
|  | Cingulate CTh |
| Spinal cord volumetry | C1 CSA |
|  | C2 CSA |
|  | C3 CSA |
|  | C4 CSA |
| qMRI | Cortical qT1 |
|  | DGM qT1 |
|  | Thalamic qT1 |
|  | NAWM qT1 |
|  | WML qT1 |
|  | Cortical MVF |
|  | DGM MVF |
|  | Thalamic MVF |
|  | NAWM MVF |
|  | WML MVF |
|  | DGM QSM |
|  | Thalamic QSM |
|  | NAWM QSM |
|  | WML QSM |
|  | NAWM ICVF |
|  | WML ICVF |
|  | NAWM MWF |
|  | WML MWF |
| Lesions | T2-WML volume |
|  | T1-WML volume |
|  | CL count |
|  | CL volume |
|  | PRL count |
|  | PRL volume |
|  | SC lesion count |
|  | SC lesion presence |
| Serum Biomarkers | sNfL |
|  | sGFAP |

*Abbreviations: CC = corpus callosum; CL = cortical lesion; CSA = cross-sectional area; CTh = cortical thickness; DGM = deep gray matter; GM = gray matter; ICVF = intracellular volume fraction; MWF = myelin water fraction; NAWM = normal-appearing white matter; PMS = progressive multiple sclerosis; PRL = paramagnetic rim lesion; QSM = quantitative susceptibility mapping; qT1 = quantitative T1; RRMS = relapsing-remitting multiple sclerosis; SC = spinal cord; sGFAP = seurm glial fibrillary acidic protein; sNfL = serum neurofilament light chain; WM = white matter; WML = white matter lesion.*

**eTable 4 - List of included predictors in Cohort 2**

| Clinical/demographic variables | Age |
| --- | --- |
|  | Sex |
|  | Disease phenotype (PMS vs RRMS) |
|  | Disease duration |
|  | Recent relapses |
| Brain volumetry | Total brain volume |
|  | GM volume |
|  | WM volume |
|  | DGM volume |
|  | Cortical volume |
|  | Thalamic volume |
|  | Cerebellar cortical volume |
|  | Cerebellar WM volume |
|  | Caudate volume |
|  | Brainstem volume |
|  | CC volume |
|  | Putamen volume |
|  | Hippocampal volume |
|  | Pallidum volume |
|  | Ventricles volume |
| CTh | Mean CTh |
|  | Frontal CTh |
|  | Parietal CTh |
|  | Temporal CTh |
|  | Occipital CTh |
|  | Insular CTh |
|  | Cingulate CTh |
| Spinal cord volumetry | C1 CSA |
|  | C2 CSA |
|  | C3 CSA |
|  | C4 CSA |
| Lesions | T2-WML volume |
|  | T1-WML volume |
|  | SC lesion count |
|  | SC lesion presence |
| Serum Biomarkers | sNfL |
|  | sGFAP |

*Abbreviations: CC = corpus callosum; CL = cortical lesion; CSA = cross-sectional area; CTh = cortical thickness; DGM = deep gray matter; GM = gray matter; ICVF = intracellular volume fraction; MWF = myelin water fraction; NAWM = normal-appearing white matter; PMS = progressive multiple sclerosis; PRL = paramagnetic rim lesion; QSM = quantitative susceptibility mapping; qT1 = quantitative T1; RRMS = relapsing-remitting multiple sclerosis; SC = spinal cord; sGFAP = seurm glial fibrillary acidic protein; sNfL = serum neurofilament light chain; WM = white matter; WML = white matter lesion.*

**eTable 5 – Characteristics of patients with and without PIRA in Cohort 1, before and after propensity score-matching**

|  | **Before propensity score-matching** | | | **After propensity score-matching** | | |
| --- | --- | --- | --- | --- | --- | --- |
|  | ***PIRA*** | ***Stable*** | ***SMD*** | ***PIRA*** | ***Stable*** | ***SMD*** |
| n | 28 | 92 |  | 28 | 28 |  |
| Age, mean, years | 51.4 | 46.5 | 0.360 | 51.4 | 52.3 | -0.069 |
| Sex (female), % | 57.1 | 58.7 | -0.031 | 57.1 | 57.1 | 0.000 |
| Disease duration, mean, years | 18.5 | 14.2 | 0.327 | 18.5 | 19.4 | -0.066 |
| Disease phenotype (RRMS), % | 57.1 | 79.4 | -0.449 | 57.1 | 50.0 | 0.144 |
| DMT category:  - mAb, %  - Orals, %  - Platform, %  - Untreated, % | 50.0  25.0  7.1  17.9 | 46.7  35.9  3.3  14.1 | 0.065  -0.251  0.151  0.097 | 50.0  25.0  7.1  17.9 | 57.1  25.0  7.1  10.7 | -0.143  0.000  0.000  0.187 |
| EDSS, mean | 4.0 | 3.2 | 0.377 | 4.0 | 3.9 | 0.034 |
| Follow-up duration, mean, years | 3.7 | 3.3 | 0.417 | 3.7 | 3.5 | 0.162 |

Abbreviations: DMT = disease-modifying therapy; EDSS = Expanded Disability Status Scale; mAb = monoclonal antibodies; PIRA = progression independent of relapse activity; RRMS = relapsing-remitting multiple sclerosis; SMD = standardized mean difference.

**eTable 6 – Time-dependent AUCs for PIRA prediction using the ridge-based risk score in Cohort 2**

| **Time interval** | **AUC (95% CI)** |
| --- | --- |
| 1 year | 0.63 (0.36; 0.91) |
| 2 years | 0.77 (0.66; 0.88) |
| 3 years | 0.81 (0.72; 0.91) |
| 4 years | 0.74 (0.63; 0.86) |
| 5 years | 0.68 (0.56; 0.80) |
| 6 years | 0.65 (0.56; 0.73) |
| 7 years | 0.61 (0.53; 0.70) |

Time-dependent area under the receiver operating characteristic curve (AUC) values for prediction of progression independent of relapse activity (PIRA) using the ridge-based risk score. AUCs were calculated at yearly time points from 1 to 7 years. Values are reported with corresponding 95% confidence intervals.

**eTable 7 – Time-dependent AUCs for PIRA prediction using the ridge-based risk score in Cohort 2**

| Time interval | AUC (95% CI) |
| --- | --- |
| 1 year | 0.53 (0.29; 0.78) |
| 2 years | 0.68 (0.55; 0.82) |
| 3 years | 0.73 (0.60; 0.86) |
| 4 years | 0.70 (0.56; 0.84) |

Time-dependent area under the receiver operating characteristic curve (AUC) values for prediction of progression independent of relapse activity (PIRA) using the ridge-based risk score. AUCs were calculated at yearly time points from 1 to 7 years. Values are reported with corresponding 95% confidence intervals.

**eFigure 1** **– Selected Predictors of EDSS ≥ 3.0 and EDSS ≥ 6.0 in Cohort 1**
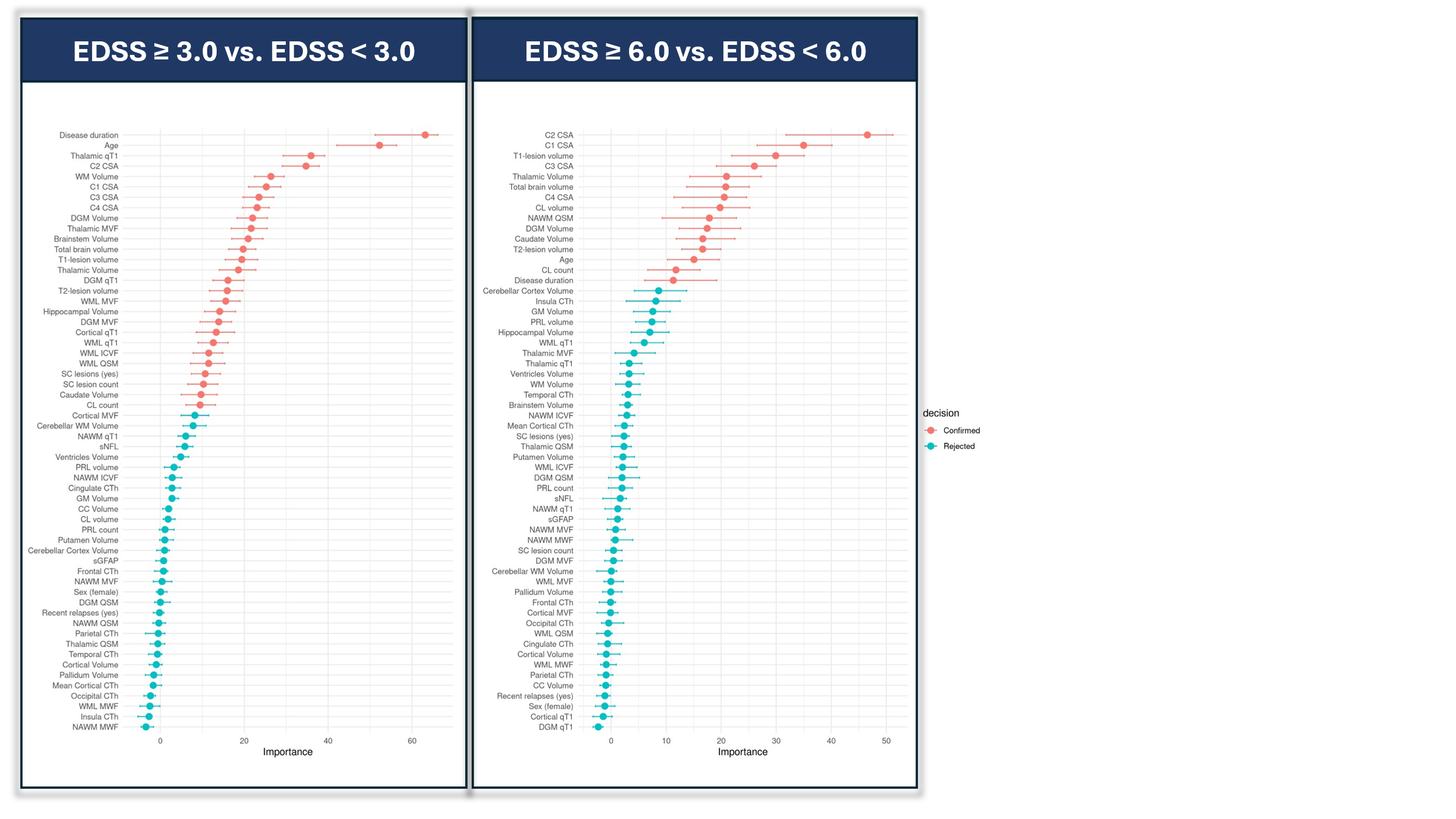


Abbreviations: CC = corpus callosum; CL = cortical lesion; CSA = cross-sectional area; CTh = cortical thickness; DGM = deep gray matter; GM = gray matter; HC = healthy control; ICVF = intracellular volume fraction; MTsat = magnetization transfer saturation; MWF = myelin water fraction; NAWM = normal-appearing white matter; PMS = progressive multiple sclerosis; PRL = paramagnetic rim lesion; QSM = quantitative susceptibility mapping; qT1 = quantitative T1; RRMS = relapsing-remitting multiple sclerosis; SC = spinal cord; sNfL = serum neurofilament light chain; WM = white matter; WML = white matter lesion.

**eFigure 2** **– Selected Predictors of SDMT (z-score based on the scores obtained in healthy controls) in Cohort 1**


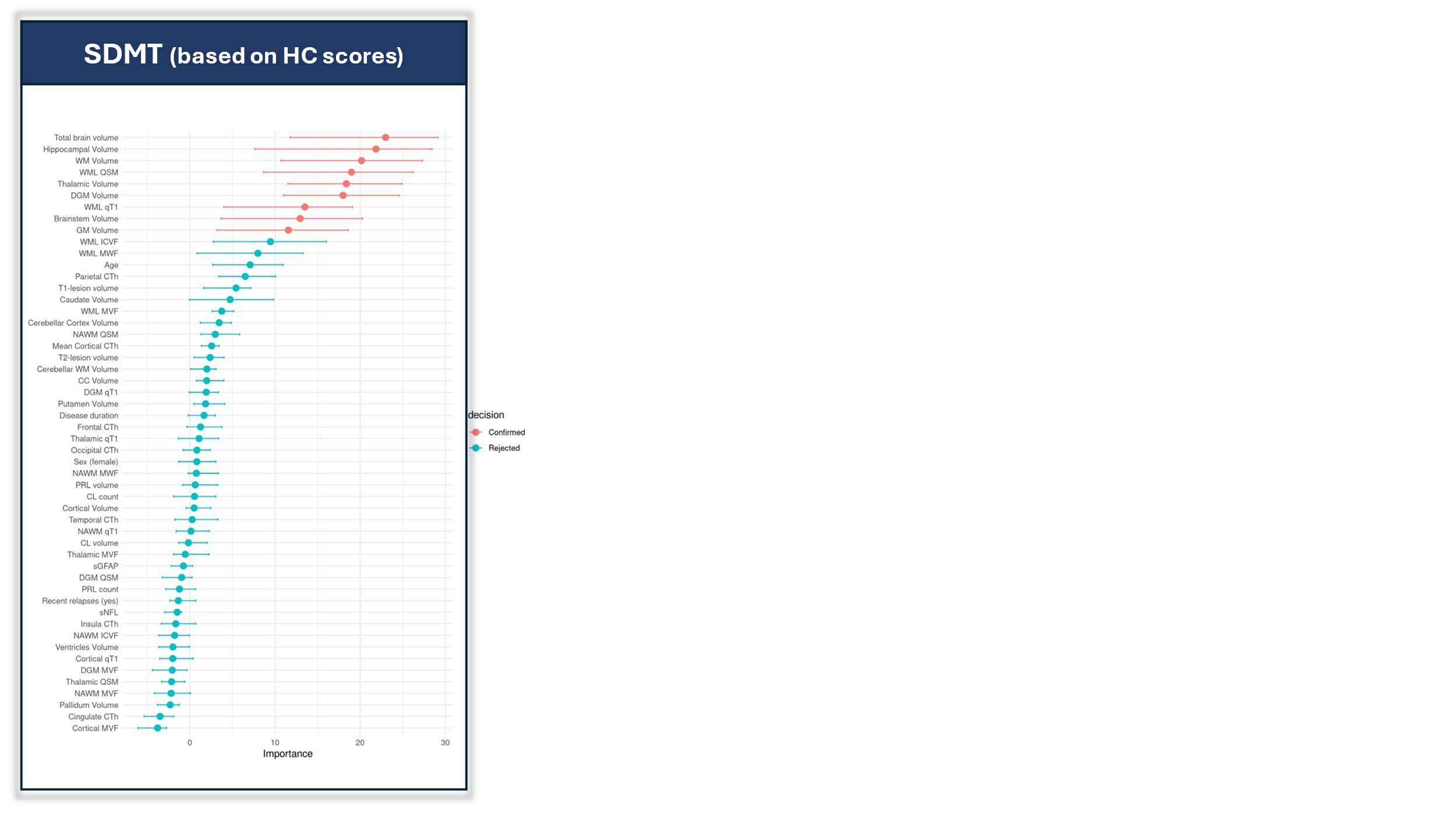
Abbreviations: CC = corpus callosum; CL = cortical lesion; CSA = cross-sectional area; CTh = cortical thickness; DGM = deep gray matter; GM = gray matter; HC = healthy control; ICVF = intracellular volume fraction; MTsat = magnetization transfer saturation; MWF = myelin water fraction; NAWM = normal-appearing white matter; PMS = progressive multiple sclerosis; PRL = paramagnetic rim lesion; QSM = quantitative susceptibility mapping; qT1 = quantitative T1; RRMS = relapsing-remitting multiple sclerosis; SC = spinal cord; SDMT = symbol digit modalities test; sNfL = serum neurofilament light chain; WM = white matter; WML = white matter lesion.

**eFigure 3** **– Selected Predictors of the Disease Phenotype (model including EDSS** **as an explanatory variable) in Cohort 1**


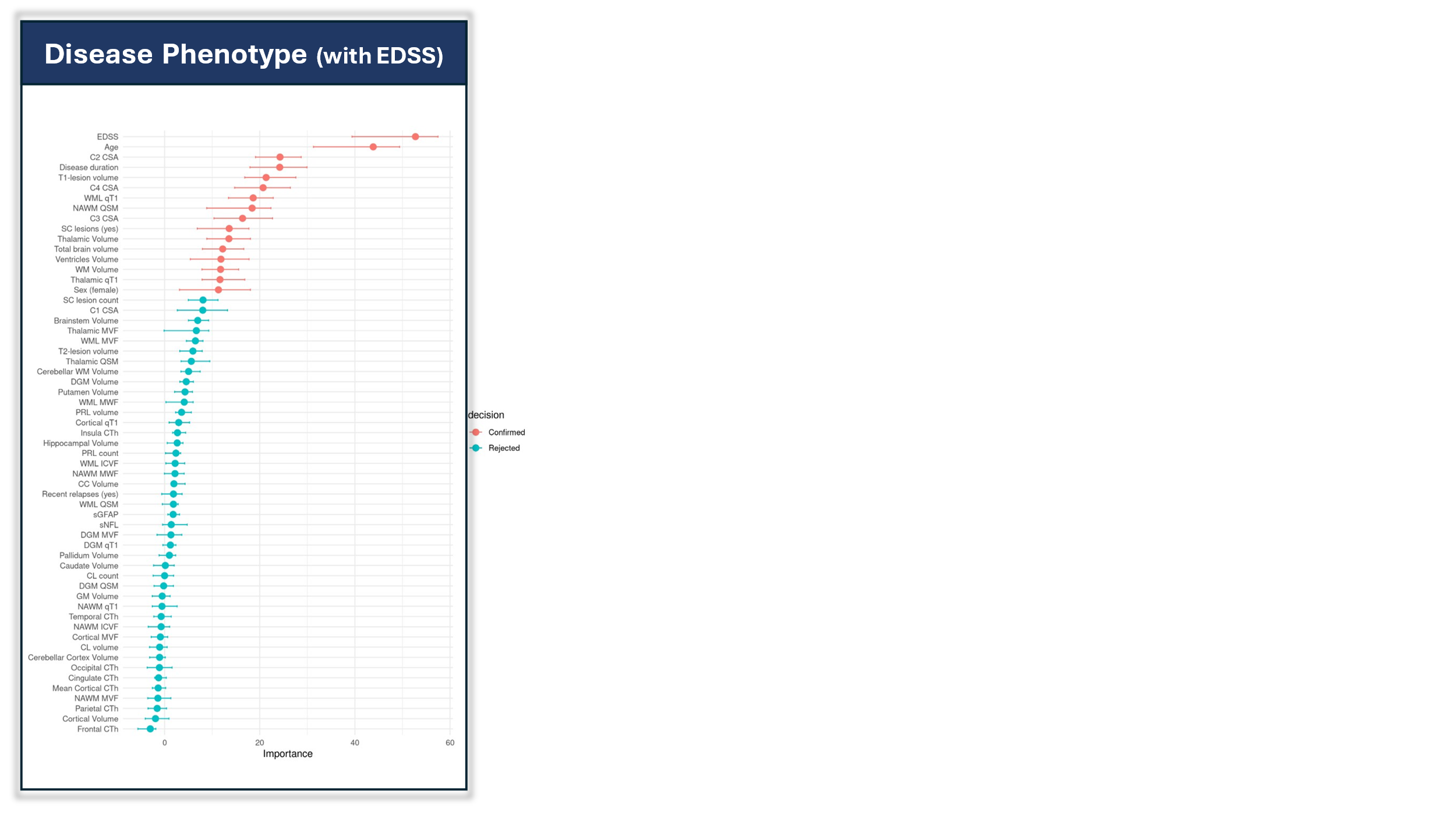
Abbreviations: CC = corpus callosum; CL = cortical lesion; CSA = cross-sectional area; CTh = cortical thickness; DGM = deep gray matter; EDSS = expanded disability status scale; GM = gray matter; HC = healthy control; ICVF = intracellular volume fraction; MTsat = magnetization transfer saturation; MWF = myelin water fraction; NAWM = normal-appearing white matter; PMS = progressive multiple sclerosis; PRL = paramagnetic rim lesion; QSM = quantitative susceptibility mapping; qT1 = quantitative T1; RRMS = relapsing-remitting multiple sclerosis; SC = spinal cord; SDMT = symbol digit modalities test; sNfL = serum neurofilament light chain; WM = white matter; WML = white matter lesion.

**eFigure 4** **– Selected Predictors of PIRMA in Cohort 1**


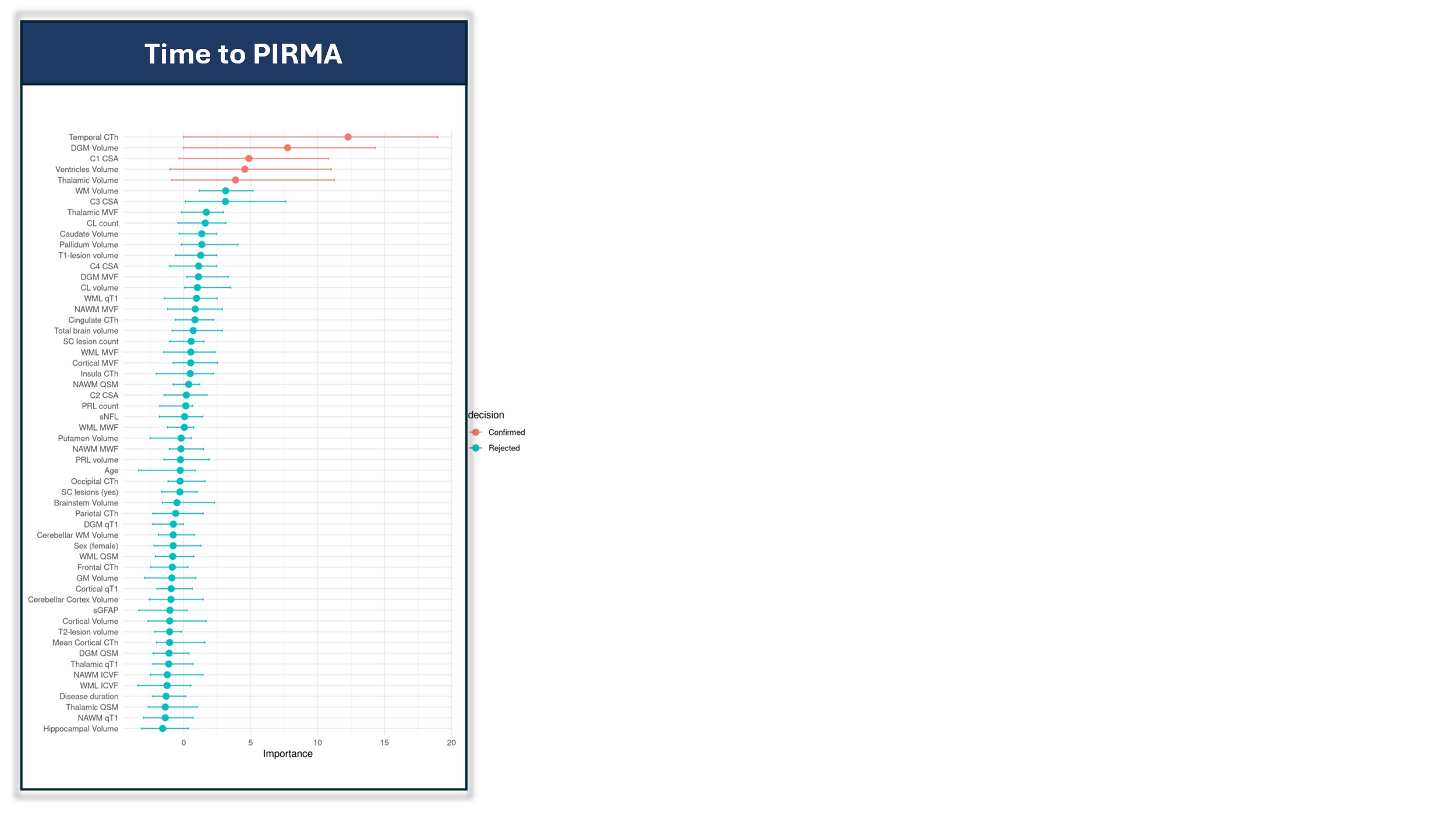
Abbreviations: CC = corpus callosum; CL = cortical lesion; CSA = cross-sectional area; CTh = cortical thickness; DGM = deep gray matter; EDSS = expanded disability status scale; GM = gray matter; HC = healthy control; ICVF = intracellular volume fraction; MTsat = magnetization transfer saturation; MWF = myelin water fraction; NAWM = normal-appearing white matter; PIRMA = progression independent of relapse and MRI activity; PMS = progressive multiple sclerosis; PRL = paramagnetic rim lesion; QSM = quantitative susceptibility mapping; qT1 = quantitative T1; RRMS = relapsing-remitting multiple sclerosis; SC = spinal cord; SDMT = symbol digit modalities test; sNfL = serum neurofilament light chain; WM = white matter; WML = white matter lesion.

**eFigure 5** **– Selected Predictors of PIRA (in patients with RRMS only) in Cohort 1**


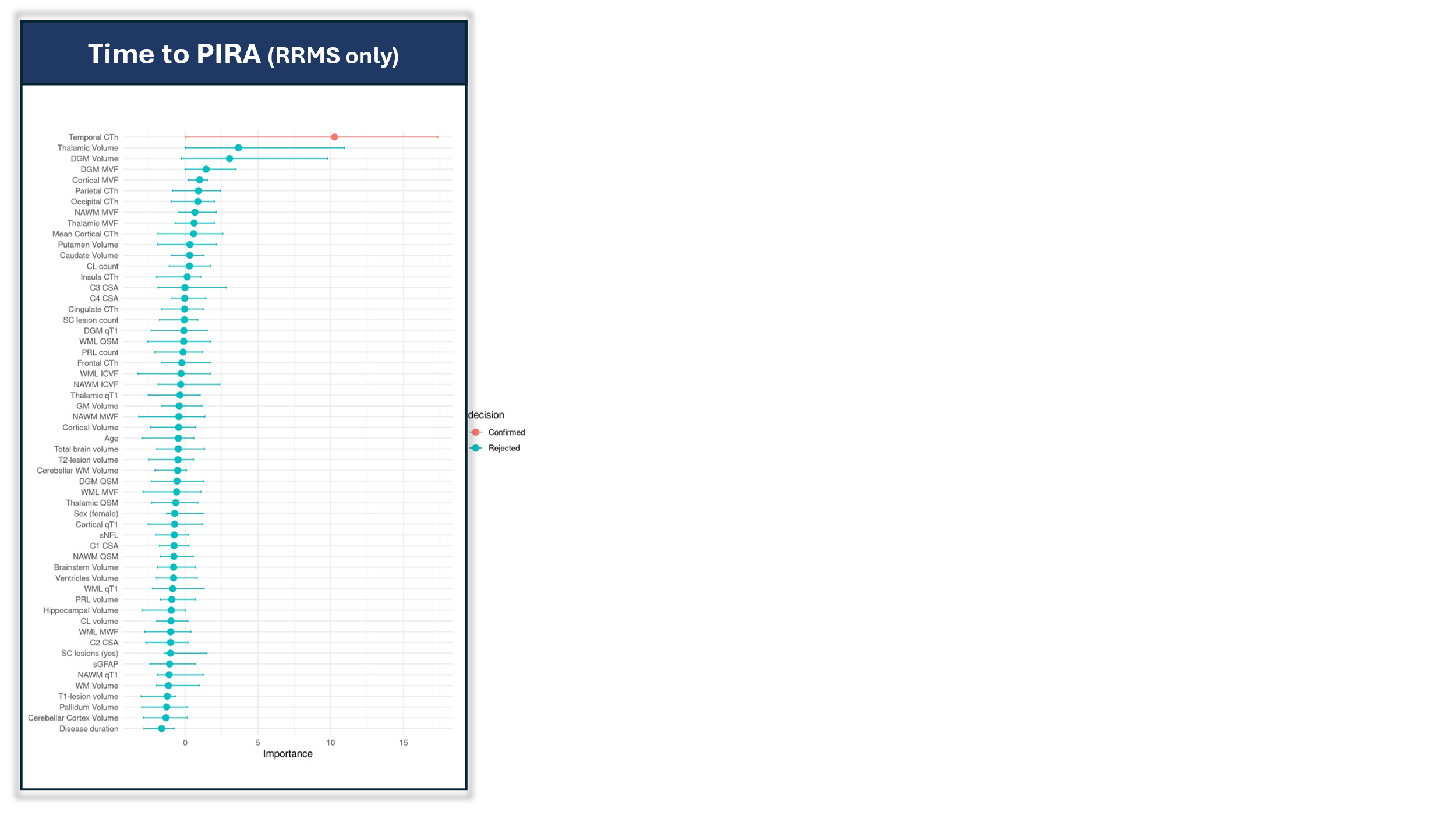
Abbreviations: CC = corpus callosum; CL = cortical lesion; CSA = cross-sectional area; CTh = cortical thickness; DGM = deep gray matter; EDSS = expanded disability status scale; GM = gray matter; HC = healthy control; ICVF = intracellular volume fraction; MTsat = magnetization transfer saturation; MWF = myelin water fraction; NAWM = normal-appearing white matter; PIRA = progression independent of relapse activity; PMS = progressive multiple sclerosis; PRL = paramagnetic rim lesion; QSM = quantitative susceptibility mapping; qT1 = quantitative T1; RRMS = relapsing-remitting multiple sclerosis; SC = spinal cord; SDMT = symbol digit modalities test; sNfL = serum neurofilament light chain; WM = white matter; WML = white matter lesion.

**eFigure 6 – SHAP dependence plots for predictors of time to PIRA identified by the Boruta model in Cohort 1**


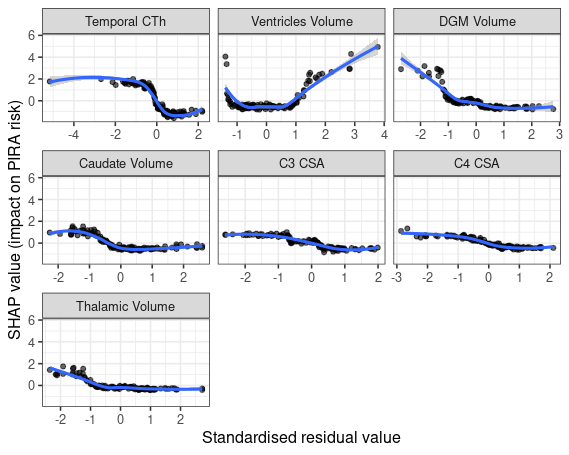


Conditional SHAP dependence plots showing the relationship between standardized residual values of biomarkers selected by the Boruta model and their contribution to the predicted risk of progression indpendent of relapse activity (PIRA) in Cohort 1. For each predictor, the x-axis represents the standardized residual value, while the y-axis shows the corrsponding SHAP value, reflecting the marginal impact of that variable on the model-predicted PRIA risk after accounting for the presence of other predictors. Positive SHAP values indicate an increased predicted risk of PIRA, whereas negative values indicate a decreased risk. Smoothed curves illustrate the average trend, highlighting potential non-linear associations between individual biomarkers and PIRA risk.
Abbreviations: CSA, cross-sectional area; CTh, cortical thickness; DGM, deep gray matter.

**eFigure 7 – Ridge Cox regression model for time to PIRA using Boruta-selected predictors**


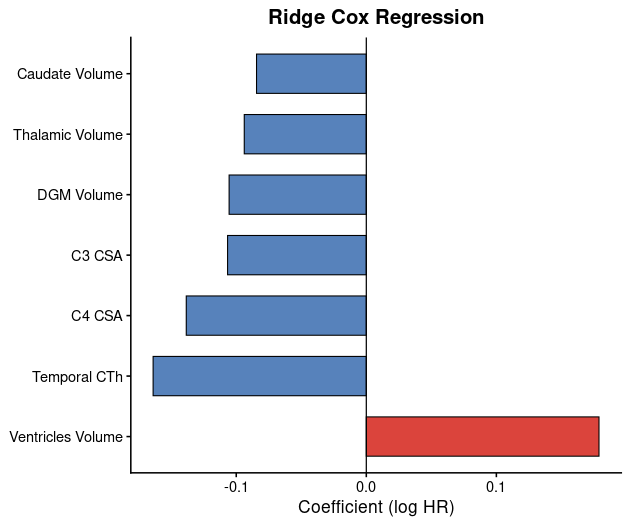


Ridge-penalized Cox regression model illustrating the association between selected imaging biomarkers and time to progression independent of relapse activity (PIRA). Bars represent standardized regression coefficients (log hazard ratios), with positive values indicating higher predicted PRIA risk and negative values indicating lower predicted risk.
*Abbreviations: CSA, cross-sectional area; CTh, cortical thickness; DGM, deep gray matter; HR, hazard ratio.*

**eFigure 8** **– Selected Predictors of EDSS ≥ 3.0 and EDSS ≥ 6.0 in Cohort 2**


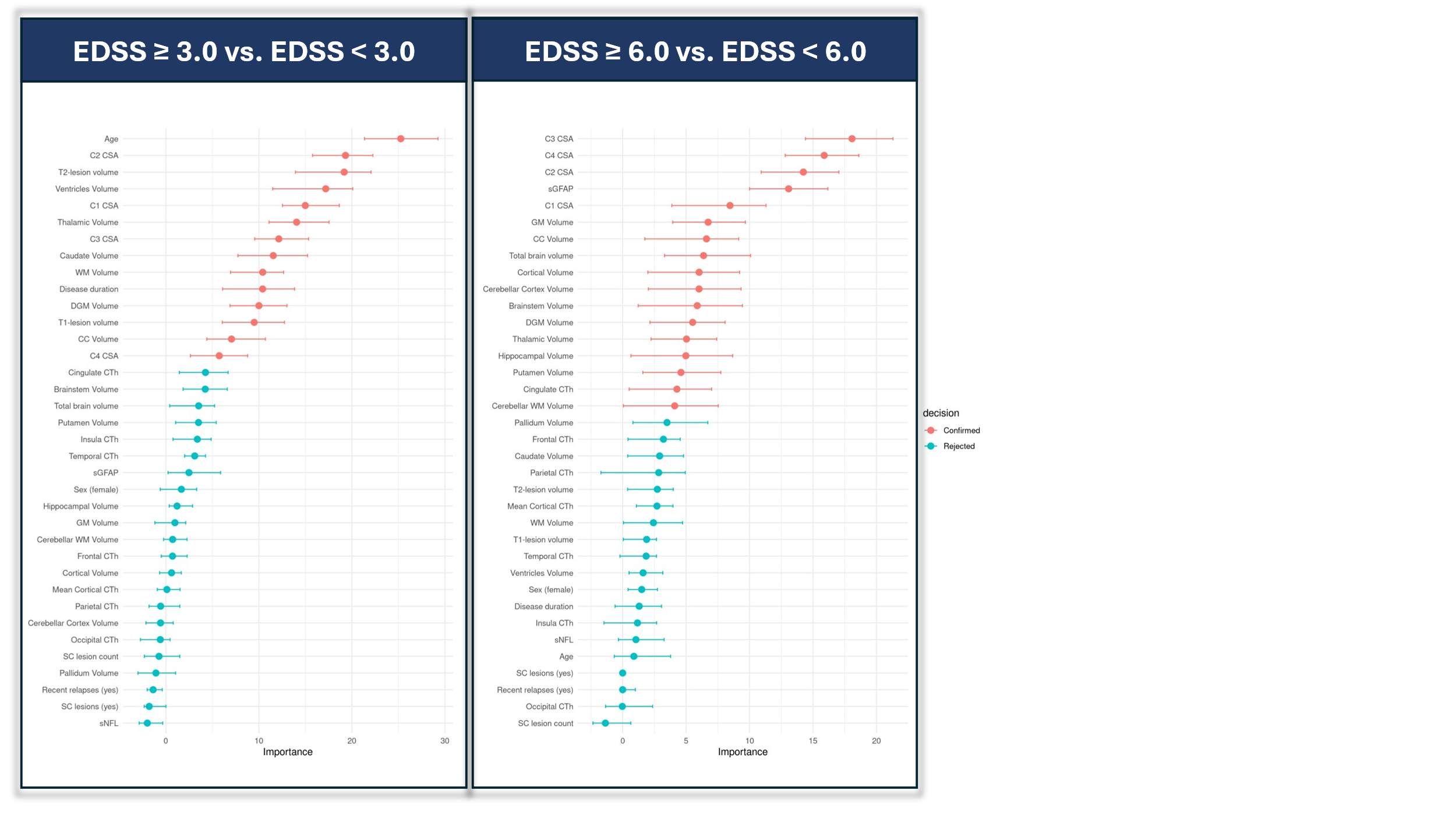


Abbreviations: CC = corpus callosum; CL = cortical lesion; CSA = cross-sectional area; CTh = cortical thickness; DGM = deep gray matter; EDSS = expanded disability status scale; GM = gray matter; HC = healthy control; PIRA = progression independent of relapse activity; PMS = progressive multiple sclerosis; PRL = paramagnetic rim lesion; RRMS = relapsing-remitting multiple sclerosis; SC = spinal cord; sNfL = serum neurofilament light chain; WM = white matter; WML = white matter lesion.

**eFigure 9** **– Selected Predictors of PIRA (in patients with RRMS only) in Cohort 2**


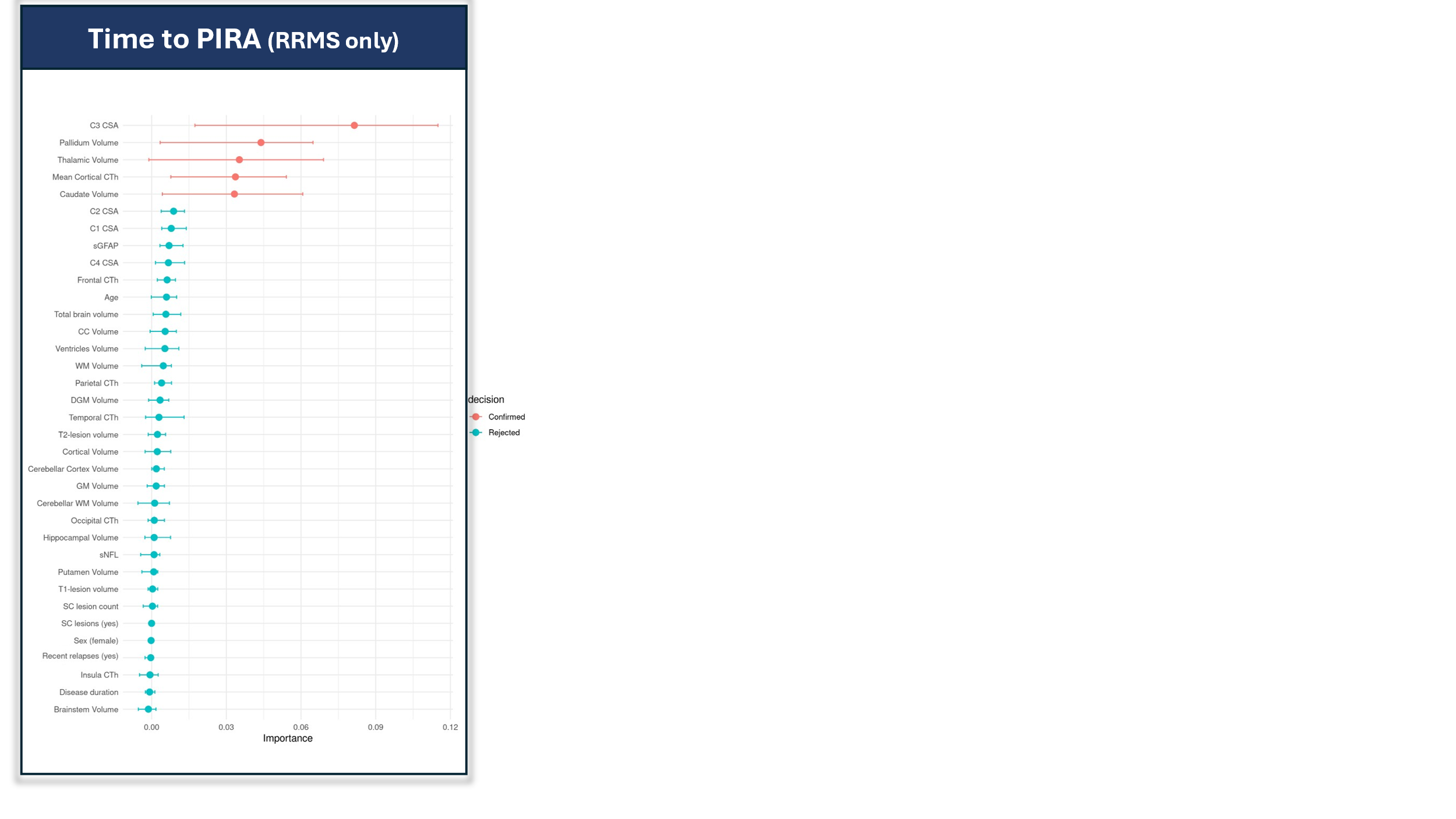
Abbreviations: CC = corpus callosum; CL = cortical lesion; CSA = cross-sectional area; CTh = cortical thickness; DGM = deep gray matter; EDSS = expanded disability status scale; GM = gray matter; HC = healthy control; PIRA = progression independent of relapse activity; PMS = progressive multiple sclerosis; PRL = paramagnetic rim lesion; RRMS = relapsing-remitting multiple sclerosis; SC = spinal cord; sNfL = serum neurofilament light chain; WM = white matter; WML = white matter lesion.

**eFigure 10** **– Selected Predictors of PIRMA in Cohort 2**


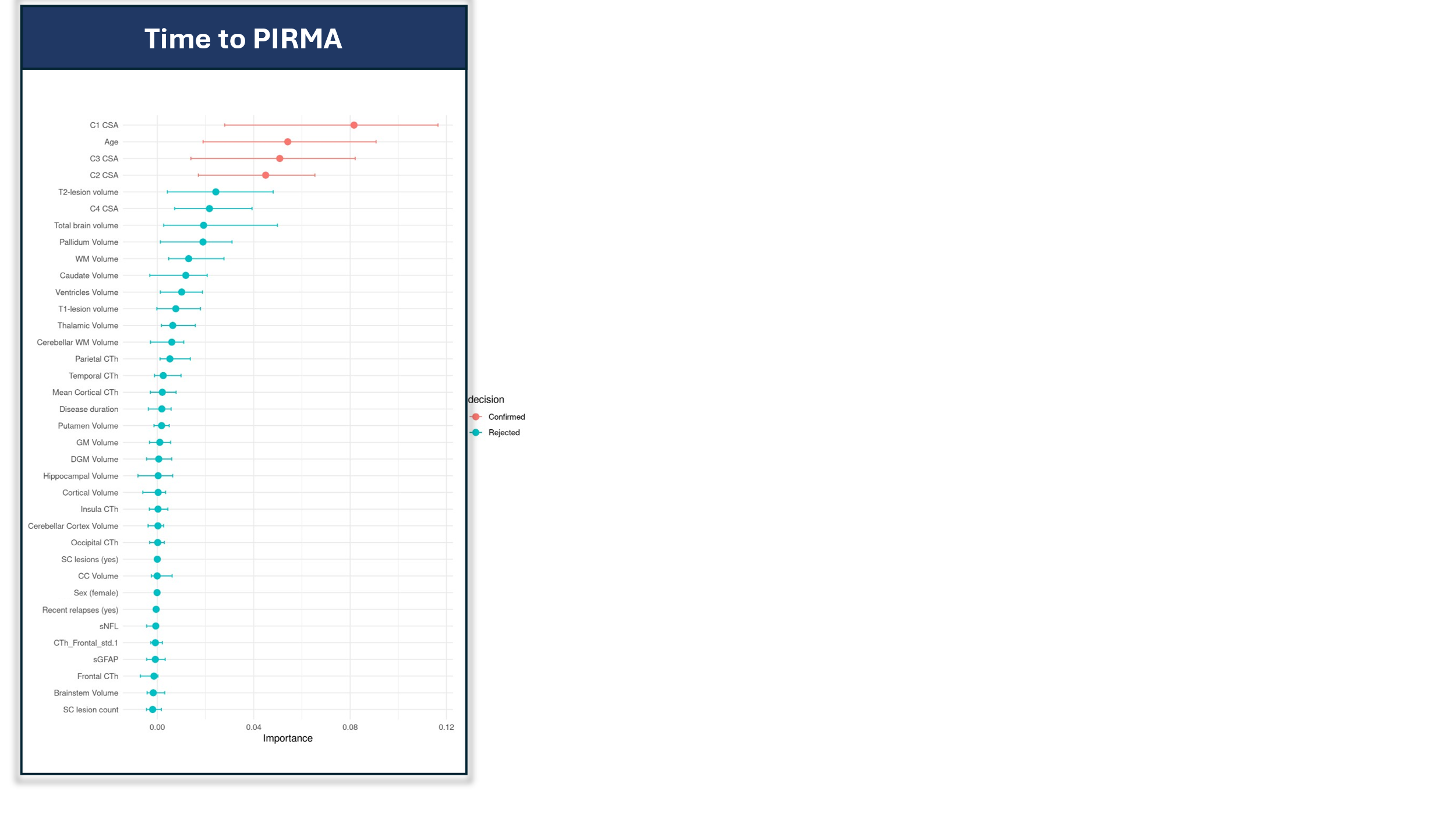
Abbreviations: CC = corpus callosum; CL = cortical lesion; CSA = cross-sectional area; CTh = cortical thickness; DGM = deep gray matter; EDSS = expanded disability status scale; GM = gray matter; HC = healthy control; PIRA = progression independent of relapse activity; PMS = progressive multiple sclerosis; PRL = paramagnetic rim lesion; RRMS = relapsing-remitting multiple sclerosis; SC = spinal cord; sNfL = serum neurofilament light chain; WM = white matter; WML = white matter lesion.**eFigure 11** **– Conditional Boruta Models in Cohort 1 – EDSS, SDMT, Disease Phenotype**


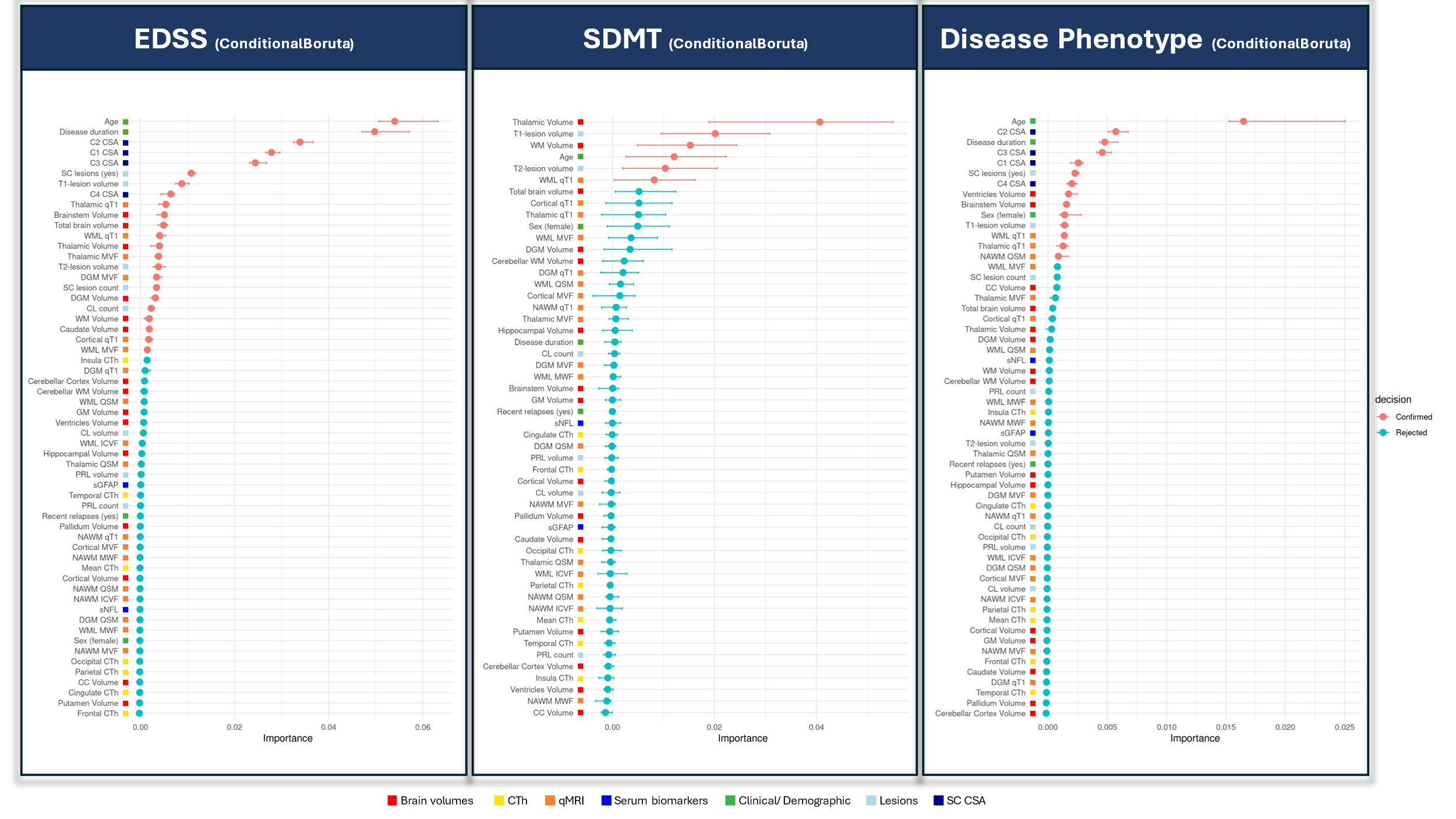


**eFigure 12** **– Conditional Boruta Models in Cohort 1 – Time to PIRA, PIRA vs. Stable**


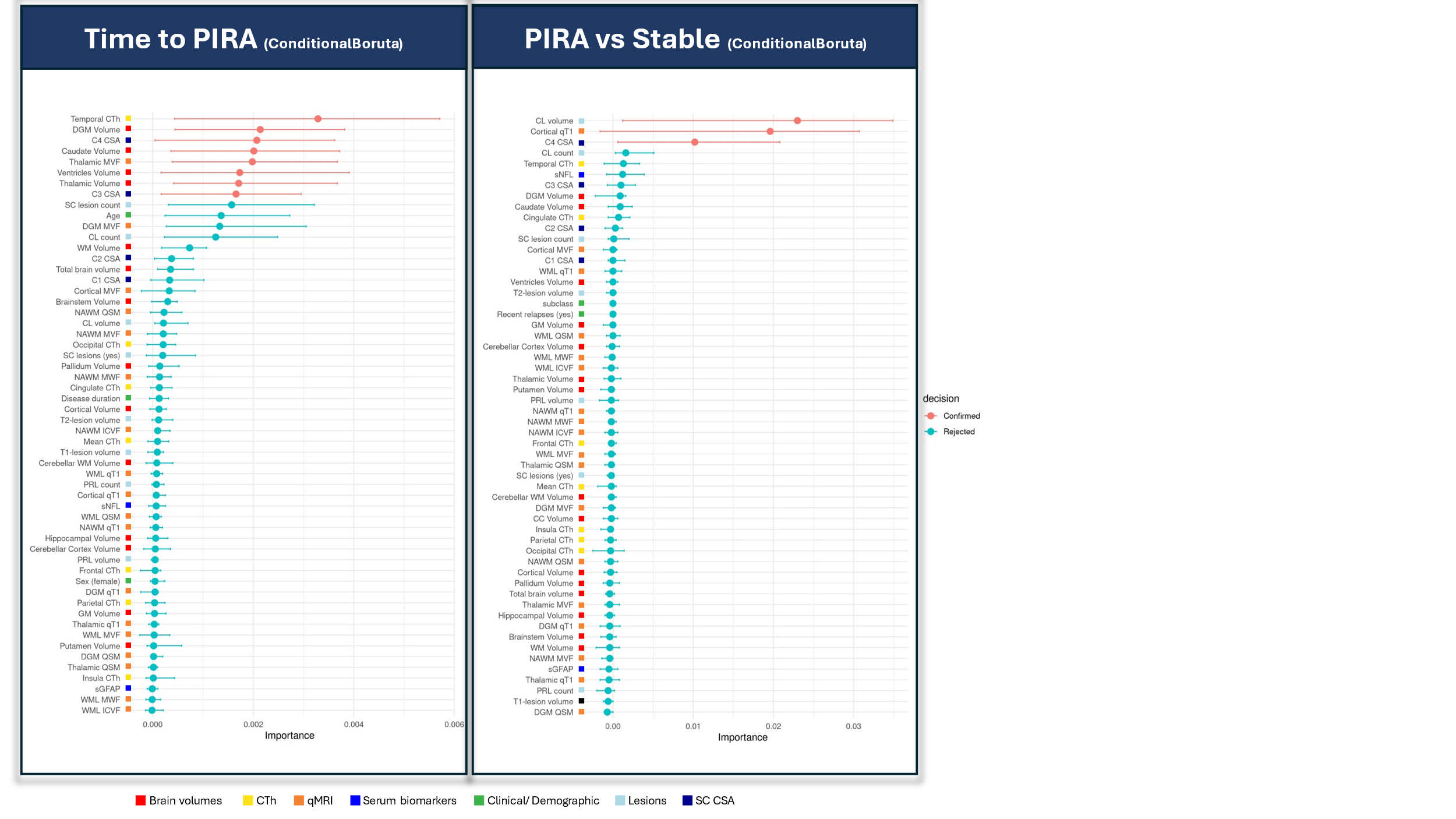


**eFigure 13** **– Conditional Boruta Models in Cohort 2 – EDSS, Disease Phenotype**


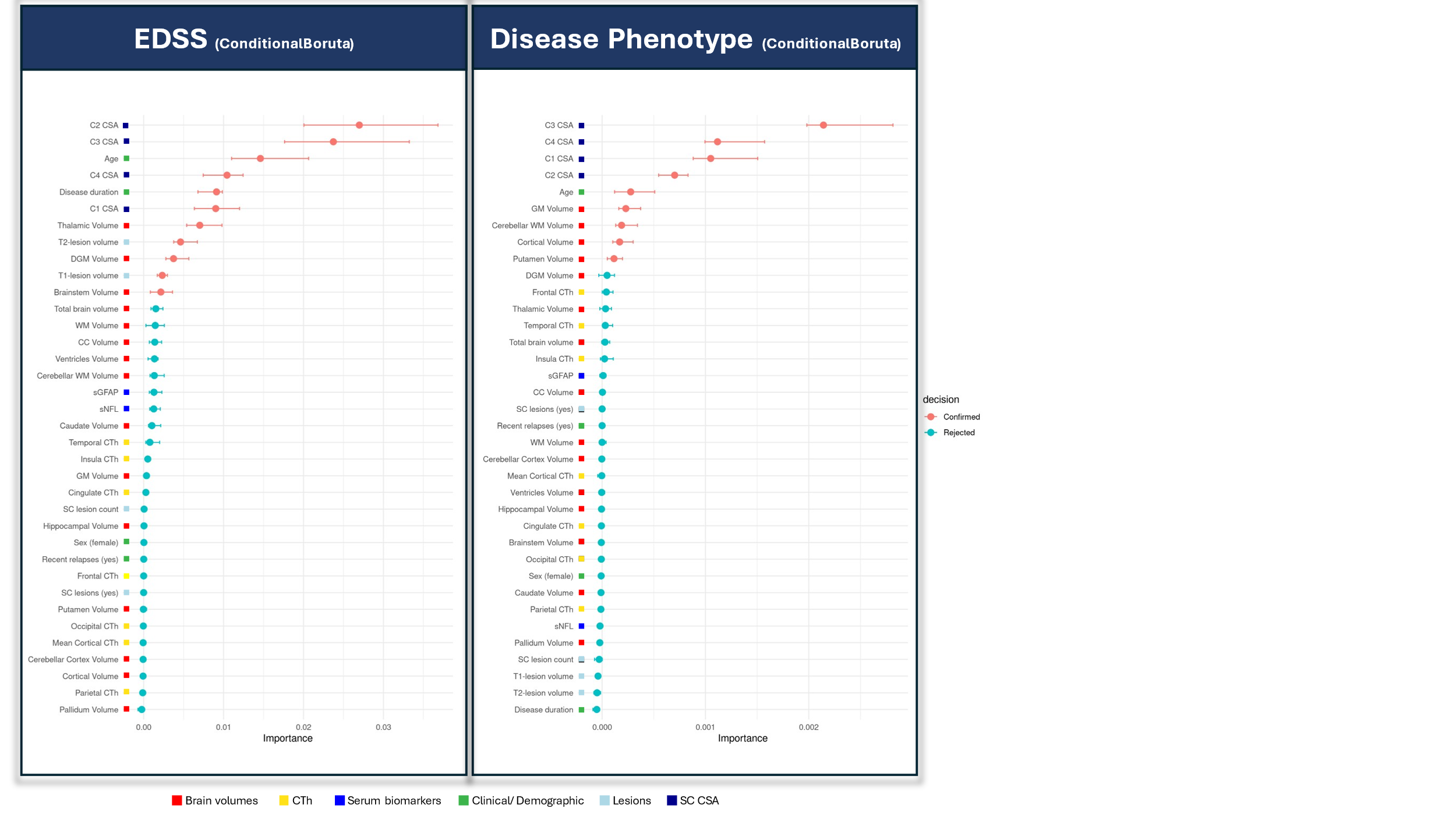


**eFigure 14** **– Conditional Boruta Models in Cohort 2 – Time to PIRA, PIRA vs. Stable**


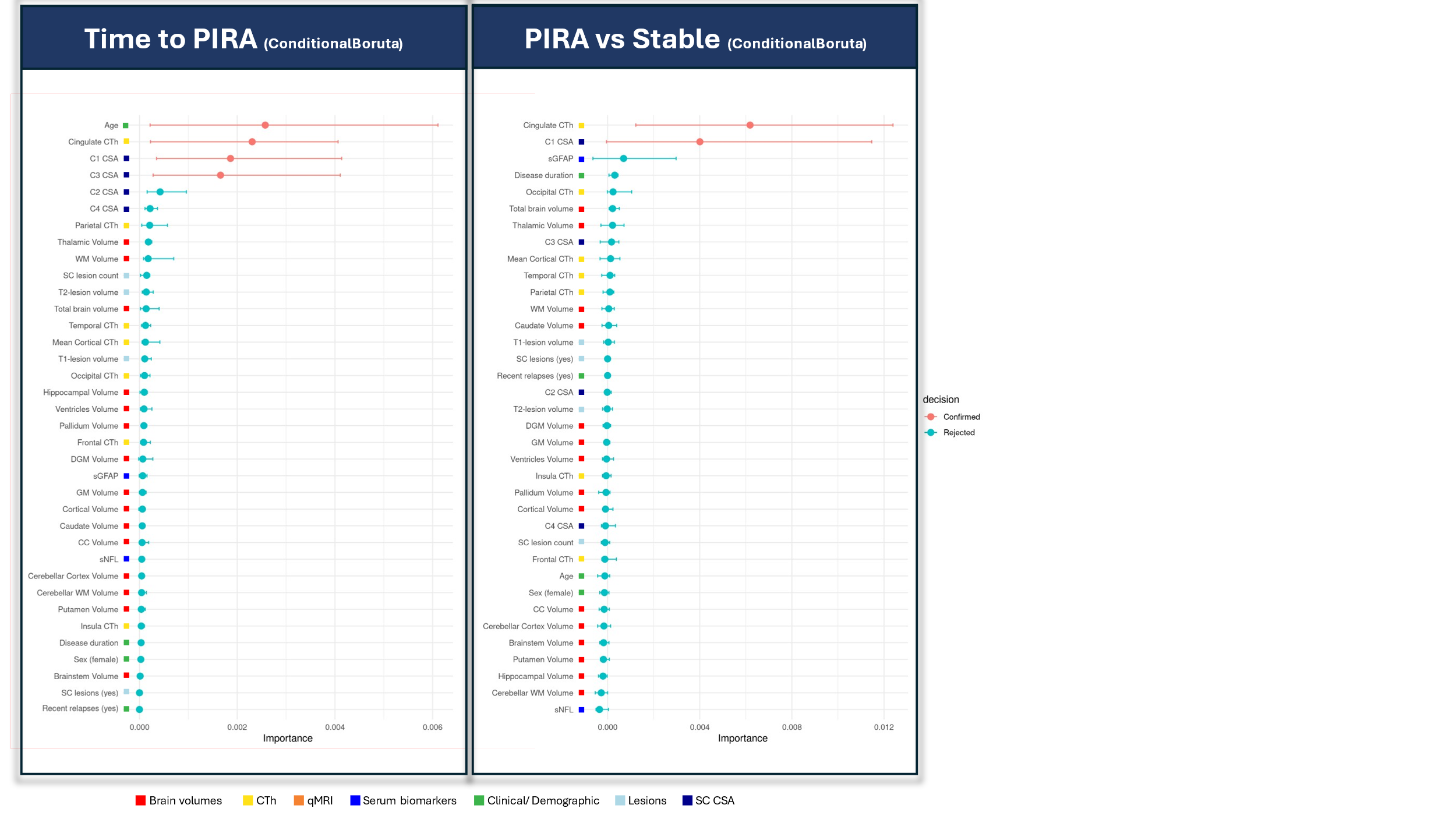


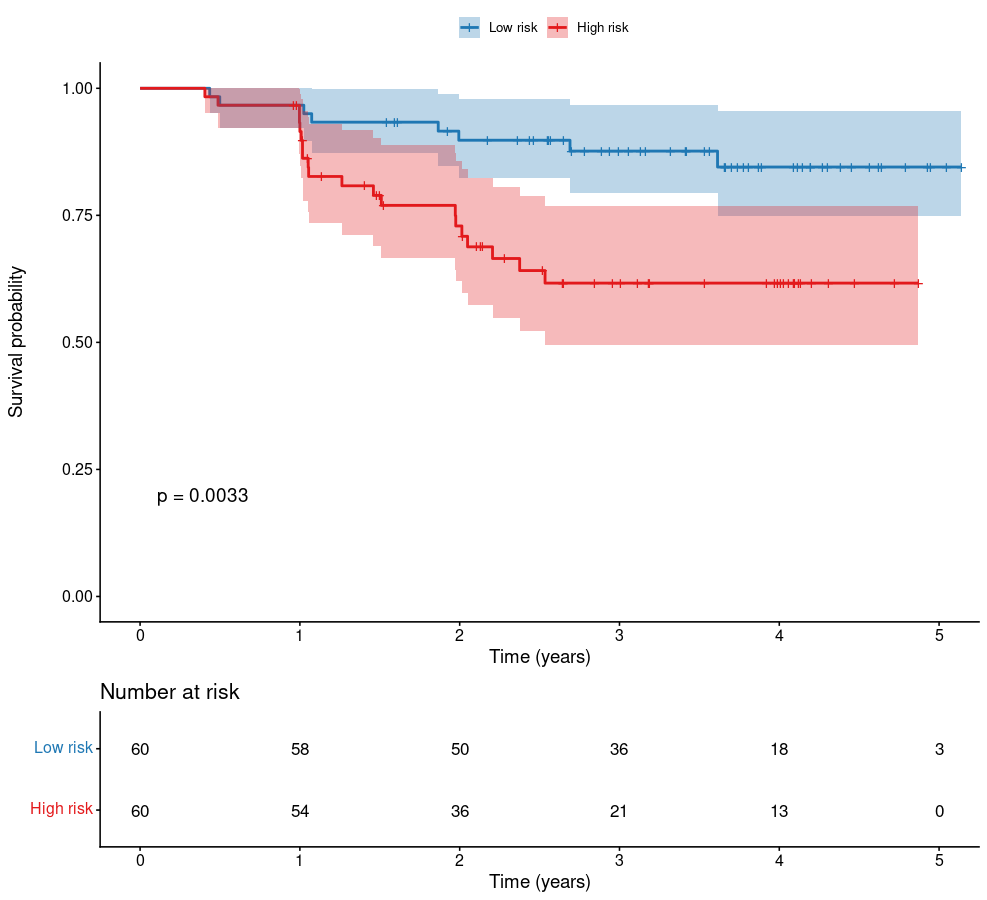
**eFigure 15 – PIRA-free survival in Cohort 1 stratified by a ridge-based clinical risk score**

Kaplan-Meier curves showing the probability of remaining free from progression independent of relapse activity (PIRA) in Cohort 1, stratified by a ridge regression-based risk score. The score was derived in Cohort 2 using a subset of clinically accessible biomarkers identified as predictors of PIRA by the Boruta analysis. Patients were stratified into high- and low-risk groups based on the median value of the risk score in Cohort 1.

**eFigure 16** **– Comparison Between Selected Predictors of EDSS in Cohort 1 and Cohort 2**


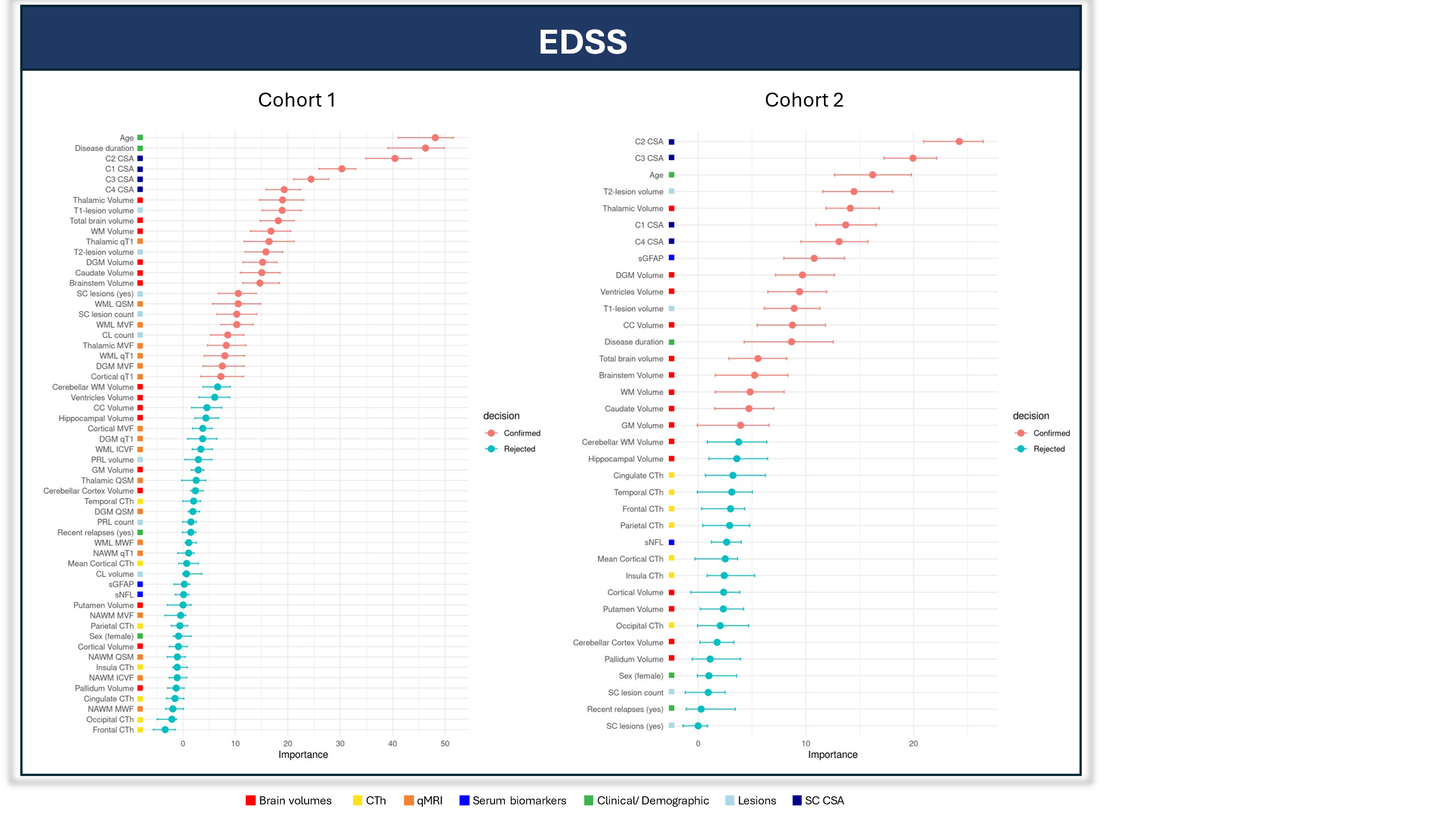


**eFigure 17** **– Comparison Between Selected Predictors of Disease Phenotype in Cohort 1 and Cohort 2**


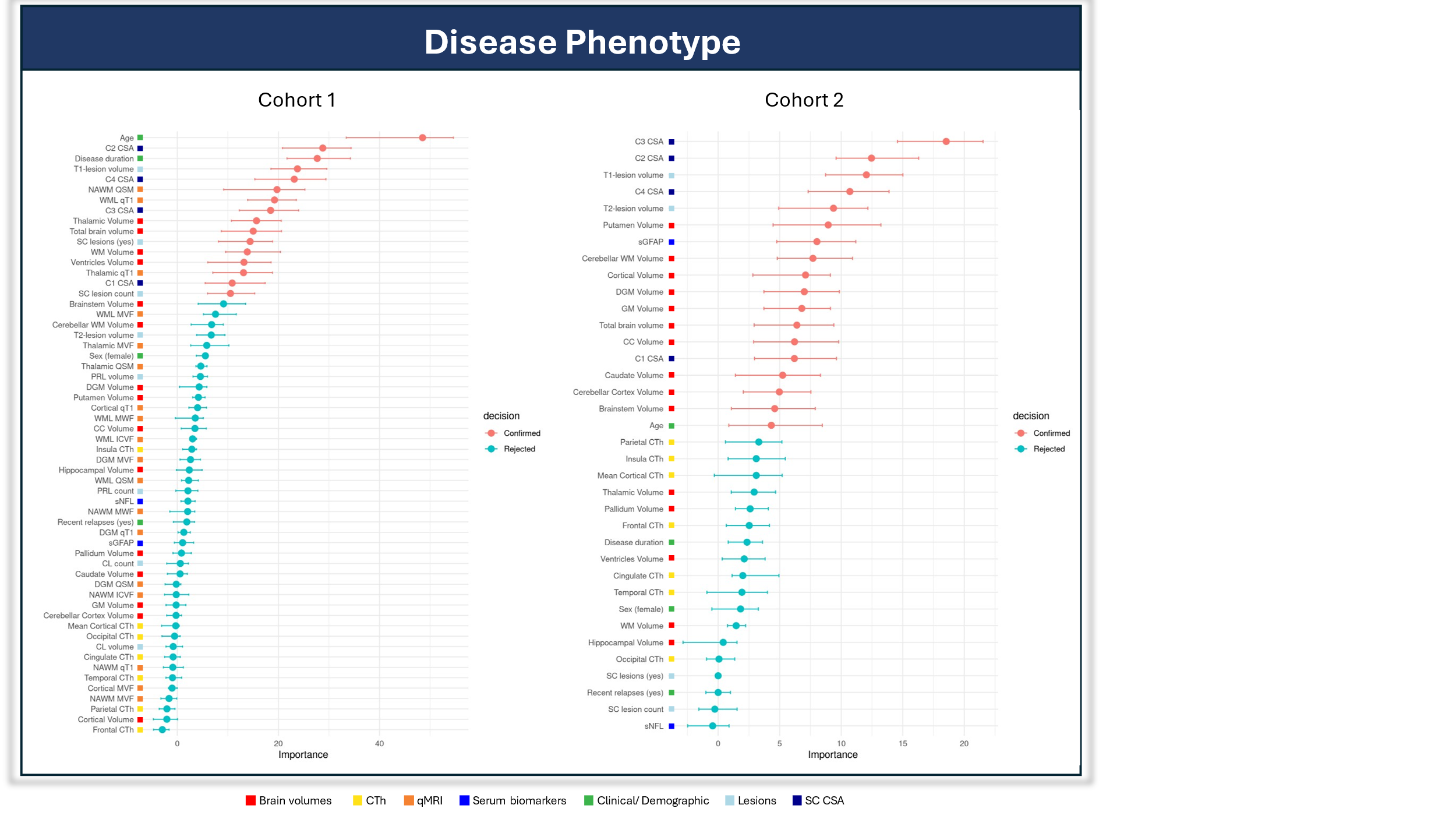


**eFigure 18** **– Comparison Between Selected Predictors of PIRA in Cohort 1 and Cohort 2**


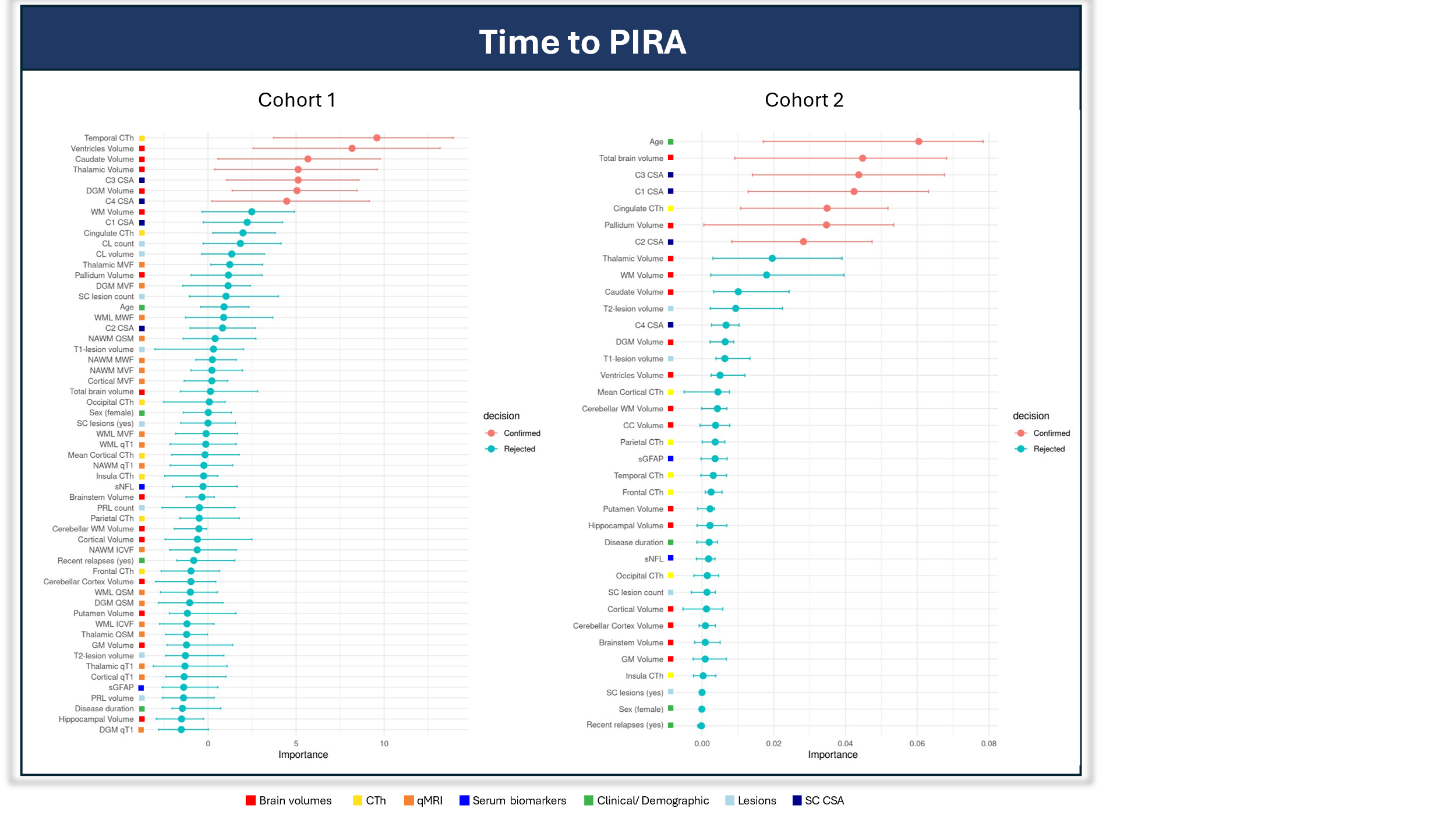

Supplement: Supplementary file 1 — Supporting File: advs73801‐sup‐0001‐SuppMat.docx. [file ADVS-13-e12946-s001.docx]
